# Mannosylated Polycations Target CD206^+^ Antigen-Presenting Cells and Mediate T-Cell-Specific Activation in Cancer Vaccination

**DOI:** 10.1021/acs.biomac.2c00993

**Published:** 2022-11-17

**Authors:** Federica Bellato, Sara Feola, Gloria Dalla Verde, Greta Bellio, Marco Pirazzini, Stefano Salmaso, Paolo Caliceti, Vincenzo Cerullo, Francesca Mastrotto

**Affiliations:** † Department of Pharmaceutical and Pharmacological Sciences, University of Padova, Via F. Marzolo 5, 35131Padova, Italy; ‡ Drug Research Program ImmunoViroTherapy Lab (IVT), Faculty of Pharmacy, Helsinki University, Viikinkaari 5E, 00790Helsinki, Finland; § iCAN Digital Precision Cancer Medicine Flagship, FI-00014Helsinki, Finland; ∥ Department of Biomedical Sciences, University of Padova, Via Ugo Bassi 58/B, 35131Padova, Italy

## Abstract

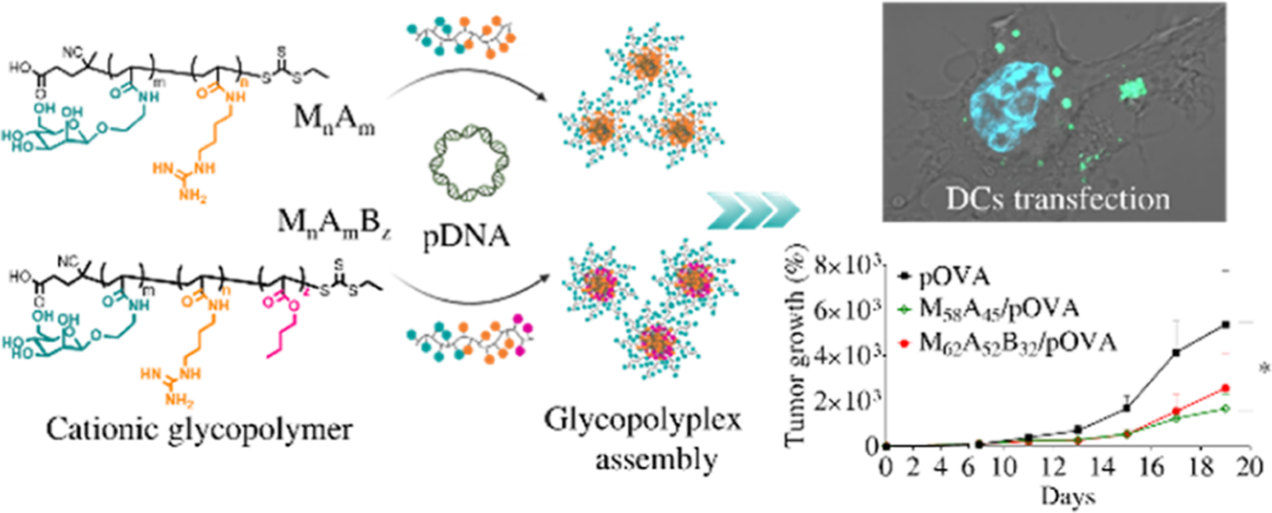

Immunotherapy is deemed one of the most powerful therapeutic approaches to treat cancer. However, limited response and tumor specificity are still major challenges to address. Herein, mannosylated polycations targeting mannose receptor- are developed as vectors for plasmid DNA (pDNA)-based vaccines to improve selective delivery of genetic material to antigen-presenting cells and enhance immune cell activation. Three diblock glycopolycations (M_15_A_12_, M_29_A_25_, and M_58_A_45_) and two triblock copolymers (M_29_A_29_B_9_ and M_62_A_52_B_32_) are generated by using mannose (M), agmatine (A), and butyl (B) derivatives to target CD206, complex nucleic acids, and favor the endosomal escape, respectively. All glycopolycations efficiently complex pDNA at N/P ratios <5, protecting the pDNA from degradation in a physiological milieu. M_58_A_45_ and M_62_A_52_B_32_ complexed with plasmid encoding for antigenic ovalbumin (pOVA) trigger the immune activation of cultured dendritic cells, which present the SIINFEKL antigenic peptide via specific major histocompatibility complex-I. Importantly, administration of M_58_A_45_/pOVA elicits SIINFEKL-specific T-cell response in C56BL/6 mice bearing the melanoma tumor model B16-OVA, well in line with a reduction in tumor growth. These results qualify mannosylation as an efficient strategy to target immune cells in cancer vaccination and emphasize the potential of these glycopolycations as effective delivery vehicles for nucleic acids.

## 1 Introduction

In recent years, the progress in understanding the basic mechanisms of the immune system and its correlation with cancer has paved the way for immunotherapy as a treatment option alternative to traditional chemotherapy.^1^ Immunotherapy marks a completely different way of treating cancer by targeting the immune system rather than the tumor itself.^2^ In this context, vaccines based on tumor neoantigens or tumor-associated antigens (TAAs)^3^ have attracted increasing attention for their ability to elicit durable antitumor immune response and tumor regression.^4^

The past decade has witnessed the rise of novel vaccination technologies based on the use of DNA or RNA,^5^ which are taken up, translated, and exposed on the membrane of transfected cells to elicit specific immune responses. Compared to the cognate antigen-based vaccines, RNA or DNA vaccines are easier to produce, more stable and safer to handle, and very cost-effective.^6^ Most importantly, their ability to provide a more natural presentation of the antigen to the immune system yields better T-cell responses, thereby eliciting stronger immunizations.^7^ Moreover, the presentation of antigens encoded by DNA-based vaccines is mediated by both class I and class II major histocompatibility complex (MHC) molecules, thus eliciting both CD4^+^ and CD8^+^ responses (i.e., humoral and cytotoxic).^8^

The efficacy, adaptability, and scalable production of DNA/RNA vaccines have been recently demonstrated in the COVID-19 pandemic,^9^ and the rising importance of DNA-based vaccines for cancer treatment is supported by the large number of ongoing clinical trials.^1c,8a,10^ Importantly, DNA/RNA vaccination strongly relies on pharmaceutical technologies, which protect nucleic acids in body fluids from degradation while improving their delivery, intracellular accumulation, and release into target cells. Indeed, as a large and negatively charged biomacromolecule, plasmid DNA (pDNA) must overcome multiple barriers; first, it must be internalized by cells, second, it should escape from the endosomal/lysosomal degradation pathway, and finally it should enter the nucleus.^11^ Moreover, insufficient gene expression^12^ and low immune system activation limit DNA vaccine application.^13^ While effective for conventional vaccination against infectious diseases, such as COVID-19, unfortunately, the success of DNA and RNA vaccines is still limited in the case of tumor therapeutic immunization.

Several types of carriers have been developed, including viral-, lipid-, and polymer-based, which come with both advantages and limitations.^4c,14^ Virus-based genetic vaccines hold safety issues, and, additionally, antiviral immune response neutralizes the vector, thus limiting repeated vaccinations. Polymers, instead, carry peculiar features making them particularly attractive.^15^ Especially, the possibility to generate polymers with customizable cationic moieties and to attach specific molecular tags allows efficient complexation of negatively charged nucleic acids, including pDNA, and the selective targeting of the carriers toward specific cell types.^16^

The C-type lectin mannose receptor (MR, CD206) being expressed on the cell surface of antigen-presenting cells (APCs), including macrophages and immature dendritic cells (DCs),^17^ has been recognized as an important target for the delivery of anti-cancer vaccines.^18^ CD206 specifically binds and internalizes a variety of glycosylated molecules, displaying a particular affinity for mannose and fucose.^19^ For this reason, CD206 plays a cardinal role in the innate and adaptive immune responses by recognizing membrane glycans and glycoproteins expressed in the outer membrane of pathogens such as bacteria and viruses. CD206 undergoes continuous cycles of endocytosis and membrane recycling, also favored by multiple rounds of ligand-induced internalization and pH-sensitive ligand dissociation.^20^ Accordingly, CD206 represents a very convenient target for cancer vaccination because it is expressed in cells specialized in antigen presentation and it is easily targetable by tagging DNA/RNA carriers with simple and biocompatible carbohydrates.

The modification of carriers with mannose tags have been proved to be a valuable strategy for a fast, selective, and efficient delivery of imaging, diagnostic, and therapeutic agents to macrophages or DCs via CD206, as indicated by diagnostic tools already approved and marketed.^21^ Zhou et al. generated micelles co-assembling pH-sensitive poly(ethylene glycol)-*block*-poly(2-(diisopropyl amino) ethyl methacrylate) and 1,2-epoxytetradecane alkylated oligoethylenimine 800 and coated them with the mannose ligand to form an acid-activatable micellar nanoparticle for the delivery of a neoantigen (i.e., OVA as model antigen) and a STING agonist, 5,6-dimethylxanthenone-4-acetic acid (DMXAA) to DCs.^16c^ Similarly, the group of Satchi-Fainaro and Florindo showed that mannosylated polylactic-*co*-glycolic acid nanoparticles efficiently deliver melanoma-associated antigens and toll-like receptor agonists to DCs.^16d^ Furthermore, the presence of mannose is expected to enhance the nuclear delivery due to the presence of lectins on the nuclear membrane,^22^ which strongly suggests that sugar residues could act as a nuclear targeting signal and may further improve the efficacy of nucleic acid processing.

In this work, we generated diblock (M_15_A_12_, M_29_A_25_, and M_58_A_45_) and triblock (M_29_A_29_B_9_ and M_62_A_52_B_32_) cationic copolymer libraries for the delivery of pDNA to APCs via reversible addition fragmentation chain transfer (RAFT) polymerization.^23^ The polymers were designed with a mannosylated (M) block to actively and selectively target CD206^24^ expressed on DCs and a polycationic agmatinyl block (A) to condense nucleic acids. In addition, a triblock library was engineered by elongating the polymer with a butyl-based hydrophobic block (B) to ideally enhance the endosomal escape properties and the transfection efficiency (TE) of this family of carriers through its membrane disruption property.^25^ Copolymers efficiently complexed model pDNA forming glycopolyplexes (GPPs) of toroid, rod, or globular shapes. Additionally, M_58_A_45_, M_29_A_29_B_9_, and M_62_A_52_B_3_ were able to prevent pDNA degradation in physiological conditions forming stable complexes which required a high concentration of competing anions to induce pDNA release. M_58_A_45_-, M_29_A_29_B_9_-, and M_62_A_52_B_3_-based GPPs efficiently transfected Chinese hamster ovary cells, either wild type (CHO) or transformed to express CD206 (CHO-CD206^+^). M_58_A_45_ and M_62_A_52_B_3_ assembled with the model antigen plasmid coding for ovalbumin (pOVA) efficiently immune-activated immortalized DCs, with different outcomes depending on the copolymer used. Importantly, M_58_A_45_/pOVA GPPs significantly controlled tumor growth *in vivo* in C56BL/6 mice bearing the melanoma tumor model B16-OVA, in which tumor-specific T-cell activation was observed, suggesting the great potential of mannosylated glycopolycations for cancer therapeutic vaccination.

## 2 Experimental Section

### 2.1 Materials

Acryloyl chloride, agmatine sulfate, carbon disulfide, ethanethiol, 4,4′-azobis(cyanopentanoic acid), *N*-hydroxyethyl acrylamide, *n*-butyl acrylate, 2,2′-azobis[2-(2-imidazolin-2-yl)propane]dihydrochloride, 1,4-dioxane, deuterated solvents, low-medium EEO agarose, glycerol, xylene cyanol FF, thiazolyl blue tetrazolium bromide, silica gel (60 Å, particle size 35–70 μm), Triton X-100, linear 25 kDa polyethylenimine (PEI_L25kDa_), analytical grade solvents, salts, cell culture media, and reagents for cell culture were obtained from Sigma-Aldrich (St. Louis, MO, USA), Fisher Scientific (Hampton, New Hampshire, USA), or Gibco ThermoFisher Scientific (Waltham, MA, USA). Butyl acrylate was purified from a phenolic inhibitor hydroquinone monomethyl ether (MEHQ) before use.^26^ α-d-Mannose pentaacetate was purchased from Apollo Scientific (Stockport, UK). GelRed nucleic acid staining solution 10,000× in water was purchased from Biotium (Fremont, CA, USA). Purified anti-mouse CD16/32, FITC anti-mouse CD11c, PerCP anti-mouse CD86, PE anti-mouse H-2Kb-bound SIINFEKL, PerCP/Cy5.5 anti-mouse CD3ε, and APC anti-mouse CD4 were supplied by BioLegend (San Diego, California, USA). FITC anti-mouse CD8 and R-PE Pro5 MHC pentamer H-2Kb SIINFEKL were obtained from Proimmune (Magdalen Centre, Oxford, UK). Mouse interferon (IFN)-γ Single-Color Enzymatic ELISPOT assay kit was purchased from ImmunoSpot (Cleveland, OH, USA).

Enhanced green fluorescence protein (pEGFP)-N3 was supplied from Addgene (Watertown, MA, USA) and amplified in XL1 blue cells. CMV-promoted pOVA was supplied by GeneScript (Leiden, Netherlands) and amplified by *Escherichia coli* bacterial cell transformation using One Shot TOP10 chemically competent *E. coli* (Invitrogen, Carlsbad, USA) and purified with a NucleoBond Xtra Midi kit (Macherey-Nagel GmbH, Düren, Germany). pDNA concentration and purity were measured on a NanoDrop One (ThermoFisher, UK) prior to complexation.

### 2.2 Analytical Methods

^1^H and ^13^C NMR spectra of monomers and polymers were acquired with a Bruker 400-AMX Ultrashield 400 MHz spectrometer (Billerica, MA, US) with samples prepared using deuterated solvents. Data were processed by MestReNova v6.0.2. Quadrupole time-of-flight (QTof) mass spectrometry was performed on a Xevo G2-XS (Waters, US). Fourier transform infrared spectrometry was performed on a Varian 640-IR FT-IR spectrometer (Agilent technologies, CA, US). Polymer molecular weight and polydispersity index (PDI, Đ) were assessed by gel permeation chromatography (GPC) analysis with Malvern Viscotek TDA302 system (Malvern, UK) equipped with a refractometer (RI), a low-angle light scattering (LALS), a right-angle light scattering (RALS), and a differential viscosimeter (Visc) and thermostated at 40 °C equipped with TOSOH G4000 (10 μm, 7.8 × 300 mm) and G3000 (7 μm, 7.8 × 300 mm) PWXL columns in series eluted with 0.4 M ammonium acetate buffer, pH 4.5. Data acquisition was performed by OmniSEC 5.1 software using the pullulan standard for calibration.

### 2.3 Typical Polymerization Conditions: Synthesis of M_*m*_A_*n*_ and M_*m*_A_*n*_B_*z*_ Block Copolymers

The synthesis and full characterization of monomers and chain transfer agents (CTAs) are detailed in the Supporting Information.

According to the procedure reported by Gody et al.^27^ and modified by our group,^28^ M_*m*_A_*n*_ diblock and M_*m*_A_*n*_B_*z*_ triblock copolymers were synthesized by fast RAFT polymerization using different CTA/monomer feed ratios and sequentially polymerizing d-mannopyranosyloxyethyl acrylamide (M) and agmatine acrylamide (A). Triblock copolymers were further chain-extended with butyl acrylate (B). To successfully perform the polymerization reaction, phenolic inhibitor MEHQ was removed from butyl acrylate, as described by Sandler and co-workers.^26^

The general polymerization procedure is here described for M_62_A_52_B_32_.

#### 2.3.1 M_62_

d-Mannopyranosyloxyethyl acrylamide (122.7 mg, 0.450 mmol) was dissolved in filtered MilliQ water (126 μL) and placed in a tube equipped with a magnetic follower. 31.5 μL of a 21.8 mg mL^–1^ 4-cyano-4-(ethylsulfanylthiocarbonylsulfanyl)pentanoic acid stock solution in dioxane (1.85 mg, 7.03 mmol) was added. The tube was then placed in an ice bath, and 4.5 μL (45 μg, 0.1406 μmol) of a freshly prepared 2,2′-azobis[2-(2-imidazolin-2-yl)propane]dihydrochloride (VA-044) initiator stock solution in filtered MilliQ water (10 mg mL^–1^) was added to the reaction mixture under stirring. The tube was sealed with a rubber septum and deoxygenated by gentle argon bubbling for 10 min. Polymerization was started by placing the tube in an oil bath preheated at 60 °C. The reaction was monitored by ^1^H NMR in DMSO-*d*_6_, analyzing samples withdrawn from the polymerization mixture at 2 h to verify that the monomer conversion was >90%. The conversion was calculated by comparing the integrals of amide group in the monomer (∼8.13 ppm) and in the polymer (7.2–7.9 ppm).

Conversion = 97%; *M*_n,th_ = 17.44 kDa; DP_th_ = 62; *M*_n(GPC, aqueous)_ = 21.1 kDa; Đ_(GPC, aqueous)_ = 1.07.

^1^H NMR (400 MHz, DMSO-*d*_6_, δ): 1.10–2.25 (m, 3H, C*H*C*H*_2 polymer backbone_); 3.10–3.76 (m, 6H, C*H*–OH_sugar_ + CH–C*H*_2 sugar_); 4.42–4.94 (m, 5H, 4× C–O*H*_sugar_ + 1× O–C*H*_anomeric_); and 7.43 (br s, 1H, N*H*).

#### 2.3.2 M_62_A_52_

Agmatine acrylamide (82.9 mg, 0.45 mmol) was dissolved in 147 μL of filtered MilliQ water in a separate tube and then transferred to the reaction vessel containing the macro-CTA M_62_ (theoretical M_62_/A molar ratio 1:64). The polymerization procedure was performed as described above until agmatine acrylamide conversion was >80%; otherwise, a second addition of the VA-044 initiator was made until that conversion percentage was reached. Polymerization was monitored by ^1^H NMR analysis of samples withdrawn from the reaction mixture and analyzing the disappearance of the vinylic proton signals (5.56, 6.06, and 6.23 ppm) as compared to the anomeric and hydroxyl protons (4.37–4.99 ppm) of M_62_ macro-CTA set as reference integrals at time 0.

Conversion = 81%; *M*_n,th_ = 27.02 kDa; DP_th_ = 114; *M*_n(GPC, aqueous)_ = 37.2 kDa; Đ_(GPC, aqueous)_ = 1.2.

^1^H NMR (400 MHz, DMSO-*d*_6_, δ): 0.94–2.35 (bm, 9H, C*H*C*H*_2 polymer backbone_ + *N*–CH_2_–C*H*_2_–C*H*_2_–CH_2 agmatine_); 2.96–3.87 (m, 6H, C*H*–OH_sugar_ + CH–C*H*_2 sugar_); 4.37–5.06 (m, 5H, 4× C–O*H*_sugar_ + 1× O–C*H*_anomeric_); and 6.88–8.11 (bm, 5H, N*H*_sugar_ + N*H*_agmatine_ + N*H*N*H*N*H*_2 guanidyl group_).

#### 2.3.3 M_62_A_52_B_32_

Finally, butyl acrylate (31.6 μL, 28.3 mg, 225 μmol) was added to the reaction vessel and polymerization (theoretical M_62_A_52_/B molar ratio 1:32) was restarted following the conditions already described until butyl acrylate conversion reached at least 90%. The polymerization was monitored by ^1^H NMR analysis of samples withdrawn from the reaction mixture by checking the disappearance of vinylic protons at 5.93, 6.17, and 6.32 ppm as compared to the anomeric and hydroxyl protons (4.37–4.99 ppm) of M_62_A_52_ macro-CTA set as reference integrals at time 0, and the chemical shift of the methylene protons from 4.10 ppm in the monomer to 3.90–4.02 ppm in the polymer. Then, the solution was diluted with deionized (DI) water and transferred into 3.5 kDa molecular-weight cutoff (MWCO) dialysis membrane and dialyzed against 5 L of DI water for 48 h with at least five water exchanges. The solution was then freeze-dried, and the polymer was recovered as a white powder (161.46 mg, 5.38 μmol, 68.45%). All polymerization intermediates and the purified M_62_A_52_B_32_ triblock copolymer were characterized by ^1^H NMR and GPC.

Conversion = 100% *M*_n,th_ = 31.12 kDa; DP_th_ = 146, *M*_n(GPC, aqueous)_ = 38.4 kDa; Đ_(GPC, aqueous)_ = 1.07.

^1^H NMR (400 mHz, DMSO-*d*_6_, δ): 0.87 (t, *J* = 7.2 Hz, 3H, C*H*_3butyl_); 1.16–1.68 (bm, 25H, C*H*C*H*_2 polymer backbone_*N*–CH_2_–C*H*_2_–C*H*_2_–CH_2 agmatine_, O–CH_2_–C*H*_2_–C*H*_2_–CH_3 butyl_); 2.99–4.09 (m, 14H, O–C*H*_2_–CH_2_–CH_2_–CH_3 butyl_ + C*H*–OH_sugar_ + CH–C*H*_2 sugar_); 4.52–5.10 (m, 10H, C–O*H*_sugar_ + O–C*H*_anomeric_); and 6.86–8.33 (bm, 10H, N*H*_sugar_ + N*H*_agmatine_ + N*H*N*H*N*H*_2 guanidyl group_).

Copolymers M_15_A_12_, M_29_A_25_, M_58_A_45_, and M_29_A_29_B_9_ were synthesized under identical conditions but using different molar ratios, as summarized in Table 1. The [CTA]/[VA-044] ratio was fixed to 1:0.02 in each addition, while the number of sequential additions varied depending on the polymer, as reported in Table S1.

**Table 1:** M_*m*_A_*n*_ Diblock and M_*m*_A_*n*_B_*z*_ Triblock Copolymer Codes, Feed Ratios, Conversion Percentage (*C* %), Final Composition, and Number-Average Molecular Weight Estimated by ^1^H NMR Analysis (*M*_n,th_), GPC (*M*_n,GPC_), and Đ

polymer code^a^	[CTA]/[M]/[A]/[B] feed ratio	*C* %^a^	[CTA]/[M]/[A]/[B]^a^ final ratio	*M*_n,th_^b^ (kDa)	*M*_n,GPC_^c^ (kDa)	Đ^c^
M_15_A_12_	1:16:16:0	94; 75	1:15:12:0	6.6	12.4	1.13
M_29_A_25_	1:32:32:0	91; 78	1:29:25:0	12.9	29.8	1.43
M_58_A_45_	1:64:64:0	91; 70	1:58:45:0	24.6	66.9	1.29
M_29_A_29_B_9_	1:32:32:9	91; 91; 100	1:29:29:9	14.8	31.1	1.16
M_62_A_52_B_32_	1:64:64:32	97; 81; 100	1:62:52:32	31.1	38.4	1.07

a Calculated by ^1^H NMR of t_end_ as described for M_62_A_52_B_32_ synthetic method.

b Calculated according to the following equation: *M*_n,th_ = [([X]_0_/[CTA]_0_) × MW_mon_ × Conv] + [MW_macro_-CTA], where MW: molecular weight; [X]_0_: initial molar concentration of the monomer; [CTA]_0_: initial molar concentration of the CTA; and Conv: conversion.

c Determined by GPC using 0.4 M ammonium acetate, pH 4.5 as the mobile phase, in a system calibrated with the pullulan standard.

### 2.4 Electrophoretic Mobility Shift Assay

GPPs were formulated by simple mixing pEGFP-N3 (0.69 μL, 100 ng, 0.0342 pmol) or pOVA (1 μL, 100 ng, 0.0246 pmol) solutions in MilliQ water with increasing amounts of polymer to achieve different nitrogen to phosphate (N/P) molar ratios (0, 1, 2.5, 5, 10, 15, and 20). The mixtures were incubated for 1 h at room temperature and then analyzed by 1% agarose gel electrophoresis at 100 V for 45 min using Tris–ammonium–ethylene diaminetetraacetic acid (EDTA) (TAE, 40 mM Tris base, 40 mM acetic acid, 1 mM EDTA) as a running buffer. The bands corresponding to pDNA were visualized under ultraviolet light after staining by immersing the gel for 30 min in 70 mL of MilliQ water containing 14 μL of 10,000× GelRed nucleic acid gel staining. The gels were imaged using a Perkin Elmer UV-Transilluminator Geliance 600 Imaging System, using Image Lab image acquisition and analysis software as the imaging system (Bio-Rad Laboratories, Headquarters, CA).

### 2.5 Heparin Displacement Assay

Displacement of pDNA by the polyanion heparin was evaluated by agarose gel. Copolymer/pEGFP complexes containing 100 ng of pDNA and prepared at the N/P ratios of 5 for M_15_A_12_ and M_29_A_29_ and 2.5 for M_58_A_45_, M_29_A_29_B_9_, and M_62_A_52_B_32_ were incubated for 15 min at 37 °C with increasing concentration of heparin (0.15–10 IU mL^–1^) and loaded into a 1% agarose gel. The conditions used for gel preparation, running, and visualization were as described in “Electrophoretic Mobility Shift Assay”.

### 2.6 Particle Size and Zeta Potential Analyses

The mean particle diameter of the GPPs and PDI were assessed by dynamic light scattering (DLS) using the Zetasizer Nano ZS (Malvern, UK) at a constant scattering angle of 173 and 25 °C. Copolymer/pEGFP complex suspensions were prepared as described in paragraph 2.3 of “Electrophoretic Mobility Shift Assay” at the N/P ratio of 5 for M_15_A_12_ and M_29_A_25_ and at the N/P ratio of 2.5 for M_58_A_45_, M_29_A_29_B_9_, and M_62_A_52_B_32_. The final pDNA concentration was fixed at 5 μg mL^–1^ for all formulations. Samples were incubated at room temperature for 1 h and then diluted to the final volume of 50 μL with 10 mM phosphate and 154 mM NaCl, pH 7.4 (PBS) and analyzed. Free copolymer solutions were analyzed at the same concentration.

The zeta potential (ZP) measurements of both free polymers and complexes were performed by laser doppler electrophoresis at a fixed polymer concentration of 0.1 mg mL^–1^ in 5 mM HEPES, pH 7.4 at the N/P ratios reported above.

### 2.7 Transmission Electron Microscopy

GPP morphology was evaluated in a negative staining mode by transmission electron microscopy (TEM, Tecnai G2 microscope (FEI)), using 1% w/v aqueous uranyl acetate staining. Samples were prepared in PBS at a final polymer concentration of 0.25 mg mL^–1^ and at the same N/P ratios used for the DLS analysis. Samples were deposited on a small holey carbon-coated support grid (400 mesh), and the solvent was allowed to dry at room temperature. The average diameter of GPPs and the percentage of spherical, rod-, or toroid-shaped complexes were evaluated by ImageJ software v.1.51 by measuring 50 individual GPPs.

### 2.8 GPP Physical and Chemical Stability in Physiological Conditions

GPPs were prepared by mixing 3.45 μL of 145 ng μL^–1^ pEGFP aqueous solution (500.25 ng, 0.171 pmol) with 0.5 mg mL^–1^ M_58_A_45_ (4.43 μL, 2.21 μg, 89.9 pmol copolymer, N/P 2.5), M_29_A_29_B_9_ (4.13 μL, 2.06 μg, 139.5 pmol, N/P 2.5), or M_62_A_52_B_32_ (4.84 μL, 2.42 μg, 77.8 pmol copolymer, N/P 2.5) aqueous solution. Samples were equilibrated at room temperature for 1 h and then diluted to 100 μL with PBS supplemented with 10% v/v FBS and incubated at 37 °C. GPP aliquots (10 μL) were withdrawn at scheduled intervals (0, 1, 3, 6, and 24 h) and (i) analyzed by agarose gel electrophoresis to assess GPP physical stability or (ii) incubated for 5 min with 1.5 μL of a 10% w/v solution of sodium dodecyl sulfate (SDS) and loaded into agarose gel to assess pDNA chemical stability within the complexes. The conditions used for gel preparation, running, and visualization were as described in “Electrophoretic Mobility Shift Assay”.

### 2.9 Cell Culture

All cell lines were grown at 37 °C in a humidified atmosphere of 5% CO_2_. CHO (wild type) and CHO-CD206^+^ (Mannose receptor-expressing CHO cells) cell lines were kindly donated by Prof. Luisa Martinez-Pomares (Faculty of Medicine & Health Sciences, University of Nottingham) and routinely cultivated in Ham’s F12/Dulbecco modified eagle’s medium (DMEM/F12) supplemented with 10% FBS, 2 mM l-glutamine, 100 U/mL penicillin, and 100 μg mL^–1^ streptomycin (DMEM/F12 complete medium). CHO-CD206^+^ cells were grown in the presence of 0.6 mg mL^–1^ of geneticin to maintain clone selection. DC2.4 cells were kindly donated by Prof. Luisa Martinez-Pomares (Faculty of Medicine & Health Sciences, University of Nottingham) and cultivated in Roswell Park Memorial Institute (RPMI) medium supplemented with 10% FBS, 2 mM l-glutamine, 100 U/mL penicillin, 100 μg mL^–1^ streptomycin, 1% non-essential amino acid (NEAA), and 50 μM β-mercaptoethanol (RPMI complete medium). JAWS II murine DCs were purchased from ATCC (Manassas, VA, USA) and grown in α-minimal essential medium (MEM) supplemented with 20% FBS, 4 mM l-glutamine, 100 U mL^–1^ penicillin, 100 μg mL^–1^ streptomycin, and 5 ng mL^–1^ granulocyte-macrophage colony-stimulating factor (GM-CSF, α-MEM complete medium). The mouse melanoma cell line B16-OVA expressing chicken OVA was kindly provided by Prof. Richard Vile (Mayo Clinic, Rochester, MN, USA) and grown using low-glucose RPMI supplemented with 10% FBS, 2 mM l-glutamine, 100 U mL^–1^ penicillin, and 100 μg mL^–1^ streptomycin. Geneticin at a concentration of 5 mg mL^–1^ was added to maintain clone selection. The mouse melanoma cell line B16-F1 was kindly provided by Prof. Véronique Préat (Université Catholique de Louvain, Louvain Drug Research Institute, Brussels, Belgium), and cells were cultivated using MEM supplemented with 10% FBS, 1% NEAA, 100 U mL^–1^ penicillin, and 100 μg mL^–1^ streptomycin. For cell experiments, GPPs were prepared in Opti-MEM added with 2 mM l-glutamine, 100 U mL^–1^ penicillin, and 100 μg mL^–1^ streptomycin (Opti-MEM). All biological assays were performed at the N/P ratio of 5 for M_15_A_12_/pEGFP and M_29_A_25_/pEGFP GPPs and at the N/P ratio of 2.5 for M_58_A_45_/pEGFP, M_29_A_29_B_9_/pEGFP, and M_62_A_52_B_32_/pEGFP GPPs.

#### 2.9.1 *In Vitro* Transfection Efficiency

GPPs were formulated as described in “Electrophoretic Mobility Shift Assay” and then diluted in Opti-MEM at 2.5 μg mL^–1^ pEGFP concentration.

##### 2.9.1.1 Flow Cytometry

CHO and CHO-CD206^+^ (1.5 × 10^4^ cells well^–1^) or DC2.4 (2.5 × 10^4^ cells well^–1^) cells were seeded in 48-well plates and were grown for 24 h. Then, the medium was replaced with 200 μL of GPP suspension in Opti-MEM and the cells were incubated for 6 h at 37 °C with 200 μL of GPPs and then washed 2 × with 100 μL of PBS and further incubated for 24 h post-transfection (24 hpt) in complete DMEM/F12 and RPMI for the CHO/CHO-CD206^+^ and DC2.4 cell lines, respectively. Afterward, the medium was discarded, and the wells were rinsed 2 × with 100 μL of PBS. Cells were detached by trypsin treatment (150 μL, 0.06% w/v solution in PBS) diluted 1:1 with PBS, fixed by addition of 100 μL of a 4% v/v paraformaldehyde (PFA) solution in PBS and stored in the dark at 4 °C until analysis. Cells were analyzed by a FACSCanto II (BD, Franklin Lakes, USA) flow cytometer and at least 1 × 10^4^ events per sample were recorded. The mean fluorescence intensity and the percentage of positive cells were detected on the FITC channel (488 nm laser, 530/30 filter). Untreated cells for each cell line served as negative controls. Data were analyzed using Flowing software v.2.5.1.

##### 2.9.1.2 Confocal Microscopy

DC2.4 cells were seeded at a density of 3 × 10^4^ cells well^–1^ on a 24-well plate containing glass dishes and were grown overnight. Cells were incubated for 6 h at 37 °C with 400 μL of GPPs, then washed 2 × with 400 μL of PBS and further incubated for 24 h. Afterward, cells were rinsed 2 × with 400 μL of PBS, fixed by incubation for 15 min with a 4% PFA solution in PBS at room temperature and rinsed 3 × with 400 μL of PBS. Nuclei were stained by incubating samples with 300 μL of a 4.5 μg mL^–1^ DAPI solution in PBS for 10 min at room temperature. Finally, glass dishes were gently rinsed 3 × with 300 μL of PBS and once with MilliQ water before being mounted on microscope slides using Mowiol aqueous mounting media.

Cells were imaged with a Zeiss confocal laser-scanning microscope (LSM 800, Zeiss, Jena, Germany) using an immersion lens with 63× magnification, with *l*_ex_ at 353 nm for nuclei detection and *l*_ex_ at 488 nm for EGFP detection. The images were then processed with ZEN2.3 blue edition software (Carl Zeiss Microscopy GmbH, Jena, Germany).

### 2.10 Activation of JAWS II Cells *In Vitro* by pOVA-Loaded GPPs and Antigen Presentation Assay

Murine DC line JAWS II was seeded at a density of 5 × 10^5^ cells well^–1^ in a 12-well plate and was grown for 24 h in complete α-MEM. Then, the medium was discarded, cells were rinsed 2 × with 1 mL of PBS and incubated for 6 h at 37 °C with 800 μL of M_58_A_45_/pOVA, M_29_A_29_B_9_/pOVA, and M_62_A_52_B_32_/pOVA GPPs formulated in Opti-MEM at 2.5 N/P ratio and 2.5 μg mL^–1^ ovalbumin-encoding plasmid (pOVA). PEI_L25kDa_/pOVA complexes at 10 N/P ratio were used as the control. Afterward, cells were rinsed with 500 μL of PBS, further incubated for 24 hpt in complete α-MEM and finally washed once with 500 μL of PBS before detachment by incubation with 500 μL of 25 mM EDTA solution for 5 min at 37 °C. Cell suspensions were collected, centrifuged, transferred to a V-bottom 96-well plate, and incubated for 10 min at 4 °C with 40 μL of Fc-receptor blocking solution (1:20 dilution in PBS of anti-mouse CD16/32, BioLegend) followed by incubation with FITC anti-mouse CD11c (1:50 dilution, 1 μL per sample, BioLegend), PerCP anti-mouse CD86 (1:40 dilution, 1.25 μL per sample, BioLegend), and PE anti-mouse H-2K^b^ bound-SIINFEKL (1:50 dilution, 1 μL per sample, BioLegend) antibodies solutions for 30 min at 4 °C. Finally, cells were centrifuged at 1500 rpm for 5 min, and the cell pellet was washed 2 × with 100 μL of PBS, fixed by incubation with 100 μL of a 4% PFA solution in PBS at 4 °C for 10 min, rinsed 2 × with 100 μL of PBS, and resuspended in 180 μL of PBS. Unlabeled samples for each condition and untreated samples were also prepared as controls. Cells were analyzed with BD LSRFortessa II flow cytometer acquiring at least 1 × 10^4^ events per sample. Data were analyzed with FlowJo software v10.2 (Becton, Dickinson and Company, Ashland, OR, USA).

### 2.11 *In Vivo* Therapeutic Vaccination Studies

All animal protocols were reviewed and approved by the experimental animal committee of the University of Helsinki (Helsinki, Finland) and the Provincial Government of Southern Finland (license number ESAVI/11895/2019). 4–6 weeks old female C57BL/6JOlaHsd immune-competent mice were purchased from Envigo (Horst, The Netherlands), used at 6–10 weeks of age, and housed in ventilated cages in clinically controlled rooms. The animals had free access to water and food, and the animal body weight was constantly monitored. All treatments were performed under isoflurane anesthesia. The treatment effect on tumor growth was evaluated by measuring the tumor volume every other day using an electronic caliper. Tumor volume was calculated according to the formula (1)
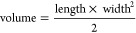
1

Body weights were assessed after tumor volume measurements.

C57BL/6 mice were divided in two groups. One group was subcutaneously injected with 3 × 10^5^ B16-OVA melanoma cells (B16-OVA tumor-bearing mice) and the other group with 1 × 10^5^ B16-F1 melanoma cells (B16-F1 tumor-bearing mice) in both flanks. Six groups of four mice per group were defined either for B16-OVA-bearing mice or for B16-F1-bearing mice: Group 1 (Mock, untreated group), Group 2 (pOVA, subcutaneous injection of pOVA), Group 3 (M_58_A_45_/pEGFP, subcutaneous injection of M_58_A_45_/pEGFP GPPs), Group 4 (M_62_A_52_B_32_/pEGFP, subcutaneous injection of M_62_A_52_B_32_/pEGFP GPPs), Group 5 (M_58_A_45_/pOVA, subcutaneous injection of M_58_A_45_/pOVA GPPs), and Group 6 (M_62_A_52_B_32_/pOVA, subcutaneous injection of M_62_A_52_B_32_/pOVA GPPs). All GPPs were prepared at an N/P ratio of 2.5 in PBS as described previously. Each mouse was subcutaneously injected at day 2, 6, 10, and 16 post tumor implantation with 100 μL containing 25 μg of pDNA for the treated groups or with PBS for the control group (Mock). The tumor progression was monitored every other day using an electronic caliper. The animals were sacrificed 21 days after tumor implantation and tumors and spleen were collected for immunological analysis.

#### 2.11.1 Immunological Analysis of Tumor Samples

B16-OVA and B16-F1 tumors from C57BL/6 mice were smashed, filtered through a 70 μm cell strainer, and cultured overnight in RPMI medium supplemented with 20% FBS. Four random tumor samples from each group were selected for tumor T-cell staining, plated in a V-bottom 96-well plate, and incubated for 10 min at 4 °C with 40 μL of Fc-receptor blocking solution (1:20 dilution in PBS of anti-mouse CD16/32). Afterward, 10 μL of Pro5 MHC Pentamer R-PE was added to each well, and the plate was incubated for 10 min at room temperature. Cells were washed once with 100 μL of PBS and then stained at 4 °C for 30 min with 50 μL of antibody staining solution containing PerCP/Cy5.5 anti-mouse CD3ε (1:40 dilution, 1.25 μL per sample), APC anti-mouse CD4 (1:40 dilution, 1.25 μL per sample), and FITC anti-mouse CD8 (1:33 dilution, 1.5 μL per sample) antibodies. Finally, cells were centrifuged at 1500 rpm for 5 min, and the cell pellet was washed twice with PBS, fixed by incubation for 10 min at 4 °C with 100 μL of a 4% PFA solution in PBS, rinsed 2 × with 100 μL of PBS and resuspended in 180 μL of PBS. Unlabeled samples were prepared for each condition and used as controls. Cells were analyzed at BD Accuri C6 Plus flow cytometer and at least 1 × 10^5^ events per sample were acquired. Data were analyzed with FlowJo software v10.2.

#### 2.11.2 IFN-γ ELISpot Assay

IFN-γ ELISpot assays were performed using a commercially available mouse ELISpot reagent set (ImmunoSpot, Bonn Germany) and 20 ng/μL of each peptide was tested *in vitro*. In brief, spleens harvested from C57BL/6 mice bearing B16-OVA and B16-F1 tumors were smashed, filtered through a 70 μm cell strainer, and cultured overnight in RPMI medium supplemented with 20% FBS. Splenocytes from each group were pooled together and treated for 5 min at room temperature with ACK buffer (155 mM ammonium chloride, 10 mM potassium bicarbonate, 0.1 mM EDTA) (red blood cells lysis buffer). Then, the splenocytes were centrifuged at 1200 rpm for 5 min, and the cell pellet was resuspended in CTL-test medium. 3 × 10^5^ cells well^–1^ were seeded in a precoated murine IFN-γ ELISpot 96-well plate (ImmunoSpot, Bonn Germany) and were stimulated with the following conditions: 1× cell activation cocktail (eBioscience, San Diego, CA, USA) (positive control), PBS (negative control), 2 μg of SIINFEKL (OVA_257–264_) peptide (for OVA specific response), and 2 μg of GP100_44–59_ peptide (for unspecific response).

After 72 h of stimulation, ELISpot plate was processed following the manufacturer’s instruction for IFN-γ detection. The plate was allowed to dry and sent to CTL-Europe GmbH for counting of the spots. Spots were counted using an ELISpot reader system (ImmunoSpot, Bonn Germany). The number of spots was normalized to the spots on cells stimulated with PBS only (negative control).

### 2.12 Statistical Analysis

Data are presented as mean ± s.d. for *in vitro* assays and as mean ± s.e.m. for *ex vivo* and *in vivo* assays. Statistical analyses were performed by using one-way or two-way analysis of variance followed by Tukey’s Multiple Comparison test. All statistical analysis were performed using GraphPad Prism (v7.0, 2018, GraphPad Software, San Diego, CA, USA). *P* < 0.05 was considered statistically significant.

## 3 Results and Discussion

### 3.1 Synthesis and Characterization of Di- and Triblock Copolymers

**Scheme 1:**
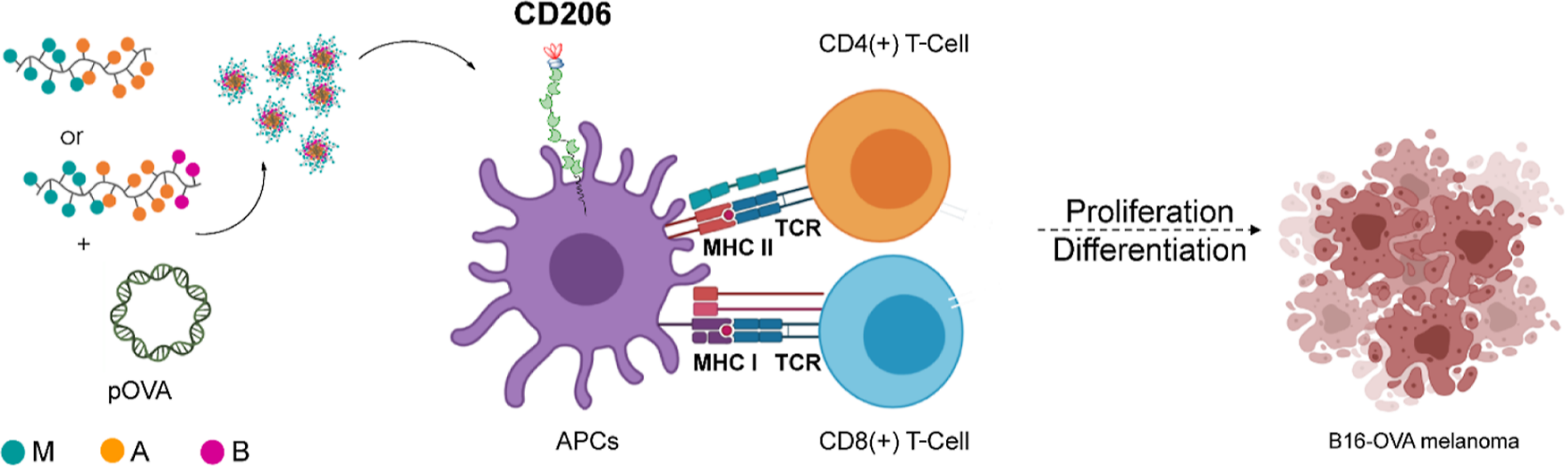
Schematic Illustration of GPP Formulation and Mechanism of Antitumoral Activity Elicited by the Developed Nanomedicine after Internalization by DCs The immunoactivating GPPs formulated by pOVA complexation with either di- or triblock mannosylated polycations actively target DCs via CD206 and deliver the pDNA to facilitate its transcription into the tumor antigen. The antigen presentation leads to CD4^+^ and CD8^+^ T-cell activation and antitumoral response that results in specific lysis of antigen-expressing tumor cells. Created with BioRender. M = mannosyl unit; A = agmatinyl unit; B = butyl unit.

With this work, we envisaged the design of a series of novel glycopolycations to efficiently complex and deliver pDNA-encoding TAA to APCs for eliciting an antitumoral immune response (Scheme 1).

Small libraries of diblock (M_*m*_A_*n*_) and triblock (M_*m*_A_*n*_B_*z*_) copolymers constituted by (i) a hydrophilic mannosylated block (M—green in Scheme 1) and (ii) a positively charged agmatine-based block (A—orange in Scheme 1) and elongated with (iii) a hydrophobic butyl-based fragment (B—pink in Scheme 1) were generated via “fast” RAFT polymerization.^23,27,28^ Both M_*m*_A_*n*_ and M_*m*_A_*n*_B_*z*_ were designed to self-assemble into polyplexes when mixed with pDNA (Scheme 1). The (M) block forms a hydrophilic outer shell that enhances colloidal stability and exposes a mannose-based antenna to selectively target CD206^+^ DCs. The (A) block constitutes a polycationic arginyl-like segment for nucleic acid condensation through the guanidine group (p*K*_a_ 12.5),^29^ which was devised to maximize the nuclear penetration of the carrier compared to other polycations, as observed by Kim and co-workers.^30^ Moreover, the length of the spacer between the polymer backbone and the guanidine group might additionally favor the nuclear disposition, as reported by Huang et al.^31^ The platform was then further developed by elongating the diblock copolymer with a hydrophobic portion (B) to foster the endosomal escape and improve the TE of this novel vehicles. Butyl-based monomers indeed can directly interact with the endosomal membrane exerting destabilizing properties, thus promoting cargo migration to the cytoplasm.^25^

**Scheme 2:**
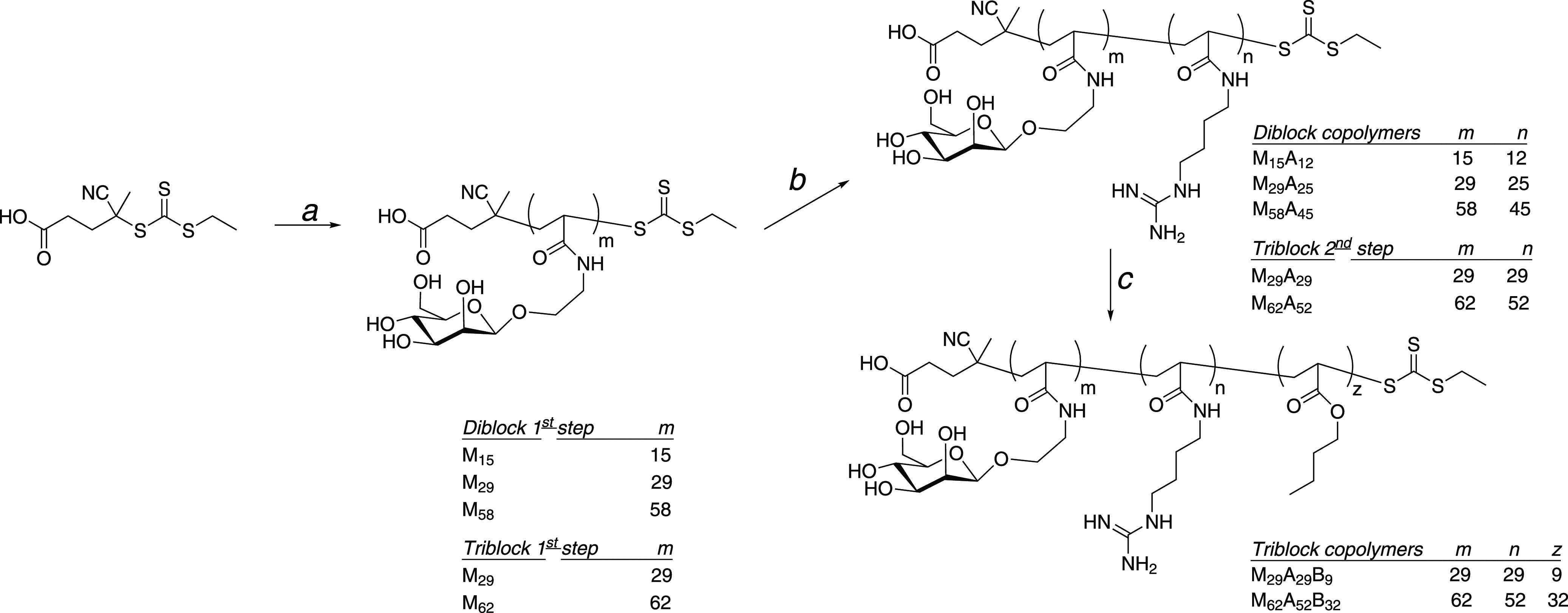
Synthesis of M_*m*_A_*n*_ Diblock and M_*m*_A_*n*_B_*z*_ Triblock Copolymers Reagents and conditions: (a) d-mannopyranosyloxyethyl acrylamide (M), VA-044, water/dioxane 80/20, Ar atmosphere, 60 °C, 2 h; (b) agmatine acrylamide (A), VA-044, Ar atmosphere, 60 °C, 2 to 4 h; (c) butyl acrylate (B), VA-044, Ar atmosphere, 60 °C, 2 to 6 h.

The 4-cyano-4-(ethylsulfanylthiocarbonylsulfanyl)pentanoic acid used as CTA was synthesized in a two-step reaction as described by Truong et al.,^32^ while d-mannopyranosyloxyethyl acrylamide (M) and agmatine acrylamide (A) monomers were synthesized according to established procedures already reported by our group and further optimized.^28b,28c,29b,29c,33^ The characterization of CTA and monomers is reported in the Supporting Information (Schemes S1–S3, Figures S1–S4). Of note, we adopted a “fast” RAFT polymerization technique,^28a^ which yielded polymers with low dispersity (Đ) and predetermined molecular weight (Table 1), and allowed to sequentially polymerize different monomers without the need of intermediate steps of polymer isolation, as it pushes monomer conversion to a very high percentage while maintaining the chain-end fidelity (Scheme 2).

To examine the effect of copolymer size and the additional presence of a hydrophobic block on pDNA complexation, pDNA protection from degradation, and TE, we generated copolymers with variable lengths (theoretical degree of polymerization (DP) in the 32–128 range for diblock copolymers) but similar M/A monomer ratios (Scheme 2, conditions a,b). Glycopolycations with DP of 64 and 128 were further elongated with the (B) hydrophobic butyl acrylate monomer (Scheme 2, conditions a–c), aiming at improving the endosomal escape properties of the new glycopolycations. The synthetic strategy was thought to yield polymers with molecular weights in the 6–30 kDa range (Table 1), thus below the threshold of renal filtration (30–50 kDa for natural polysaccharides and polyethylene glycol)^34^ to avoid body accumulation.^35^ Molecular weights measured by gel permeation chromatography (GPC) slightly deviated from the theoretical values probably due to their different hydrodynamic volume compared to the pullulan standard used for the calibration.^36^ The ^1^H NMR spectra of di- and triblock copolymers after purification and lyophilization are reported in the Supporting Information (Figures S6–S10).

### 3.2 GPP Formulation and Characterization

**Figure 1:**
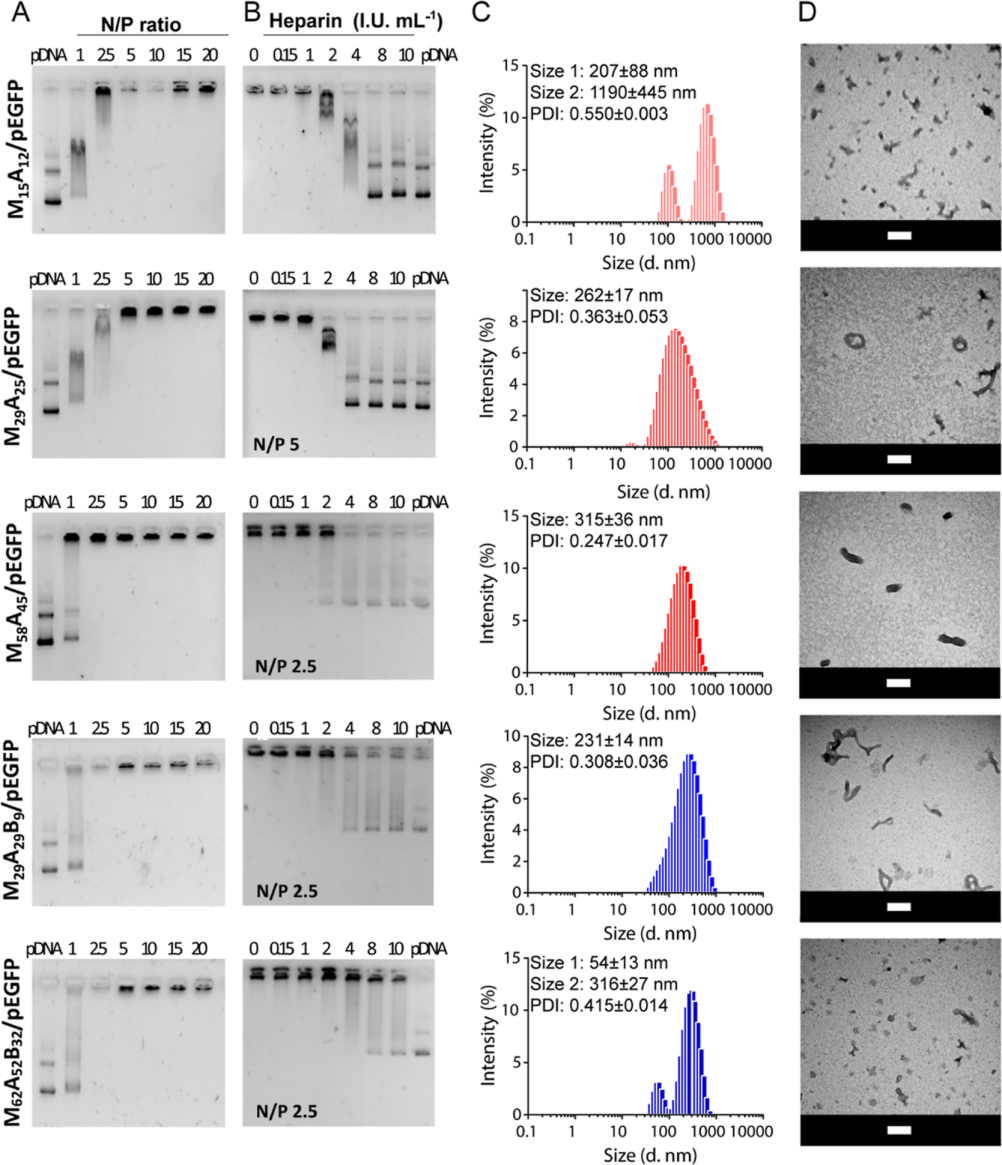
Characterization of copolymer/pEGFP GPPs. (A) Agarose gel retardation assay of M_15_A_12_/pEGFP, M_29_A_25_/pEGFP, M_58_A_45_/pEGFP, M_29_A_29_B_9_/pEGFP, and M_62_A_52_B_32_/pEGFP GPPs formulated in the 1–20 N/P ratios range. After 1 h incubation, the nanoparticles were analyzed by 1% agarose gel electrophoresis. (B) Heparin displacement assay, (C) hydrodynamic diameter measured by DLS, and (D) TEM images obtained with 1% uranyl acetate negative staining of M_15_A_12_/pEGFP and M_29_A_25_/pEGFP GPPs prepared at the N/P ratio of 5 and M_58_A_45_/pEGFP, M_29_A_29_B_9_/pEGFP, and M_62_A_52_B_32_/pEGFP GPPs prepared at the N/P ratio of 2.5. For displacement assays, GPPs were incubated for 15 min at 37 °C in the presence of increasing amounts of heparin (0–10 IU mL^–1^) and then analyzed by 1% agarose gel electrophoresis. Scale bar: 100 nm.

The ability of M_*m*_A_*n*_ and M_*m*_A_*n*_B_*z*_ block copolymers to condensate nucleic acids was examined using model pDNA, either encoding for pEGFP or for chicken ovalbumin (pOVA). The copolymer condensation capacity and the size of the formed complexes with therapeutic nucleic acid are key parameters affecting the interaction with cells and the TE, which in turn dictate the therapeutic outcomes. pEGFP or pOVA were mixed with diblock or triblock copolymers at increasing N/P ratios (1–20 range). The electrophoretic mobility shift assays (EMSA) indicated a strong binding of pEGFP for all copolymers (Figure 1A). However, M_58_A_45_, which has the highest DP among the diblock copolymers, and M_29_A_29_B_9_ and M_62_A_52_B_32_ triblock copolymers were the most efficient and complexed pEGFP at a relatively low N/P value of 2.5. Differently, M_15_A_12_ and M_29_A_25_ diblock copolymers, which have a lower DP, required at least an N/P ratio of 5 to properly condense the pDNA. Similar results were obtained when M_58_A_45,_ M_29_A_29_B_9_, and M_62_A_52_B_32_ were complexed with pOVA (Figure S18), where an N/P ratio in the 1–2.5 range was sufficient to fully condense the nucleic acid for the triblock copolymers. Altogether, these results indicate that for diblock, there is an inverse relationship between copolymer size and N/P ratio, that is, the higher the molecular weight, the lower the N/P ratio required for full pDNA complexation, as expected from previous reports.^37^ Moreover, they also suggest that the addition of the third hydrophobic block might represent a strategy to enhance the pDNA binding capacity of diblock polycations.

A critical parameter for polyplexes is their rate of DNA unpackaging, that is, the tendency of the complexed nucleic acids to be released from the polymers. This feature must be tightly balanced to avoid either a premature segregation in body fluids before cell entry or a poorly efficient intracellular DNA discharge, both leading to inefficient gene transfer. To examine this property, we mixed our copolymers with increasing amounts of heparin sulfate, a polyanionic molecule present in blood that competes with pDNA for binding to the polycationic moieties of the carriers. Concentrations in the 1–2 IU mL^–1^ range, which are roughly 10 times higher than the physiological concentration of heparin found in blood,^38^ were required to induce initial pEGFP displacement for all copolymers (Figure 1B). This strongly supports the stability of our GPPs in physiological conditions after administration. At the same time, these results also suggest that the addition of a hydrophobic block in triblock copolymers, while facilitating the assembly of GPPs by decreasing the N/P ratio required for full complexation, does not affect pDNA release. On the contrary, M_15_A_12_ and M_29_A_25_ diblock copolymers showed lower stability, releasing more massively the complexed pEGFP as compared to the other copolymers at 2 IU mL^–1^ heparin concentration. According to these results and considering that carriers exposing in their outer shell free positive charges display unspecific cell association^39^ and toxicity^40^ due to enhanced membrane-penetrating property, we decided to consider for the following experiments the GPPs with N/P ratios of 5 for M_15_A_12_ and M_29_A_25_ and 2.5 for M_58_A_45_, M_29_A_29_B_9_, and M_62_A_52_B_32_. Importantly, the mannosylated outer corona and the relatively low N/P ratios required for full complexation successfully neutralized the copolymer positive charges as indicated by the decrease of the zeta potential observed before and after complexation (Figure S11), with values in the range from +5.5 to +9.4 mV for all copolymers/pEGFP complexes, range considered as cell-inert.^41^

Size characterization by DLS analysis of GPPs prepared in PBS revealed the presence of at least two populations for M_15_A_12_/pEGFP (267 ± 88 nm and 1190 ± 445 nm) and M_62_A_52_B_32_/pEGFP (54 ± 13 nm and 316 ± 27 nm) complexes and a single population in the 230–315 nm size range for the others (size and PDI values are reported in Table S2). The PDI was in the 0.247–0.550 range for all GPPs, thus indicating the co-existence of particles of variable size. This was confirmed by TEM analysis in which various shapes were detected consisting of a mixture of toroidal, rod, and globular GPPs for M_29_A_25_/pEGFP, M_58_A_45_/pEGFP, and M_29_A_29_B_9_/pEGFP (Figures 1D and S12). Conversely, M_62_A_52_B_32_/pEGFP GPPs were characterized by bunch-like shapes, whereas M_15_A_12_/pEGFP complexes formed large aggregates due to inter-polyplex association. Of note, the preponderant morphology of GPPs could shift from elongated toroid (ring shape) or rod to globular, depending on the length of the cationic block (e.g., from M_29_A_29_B_9_/pEGFP to M_62_A_52_B_32_/pEGFP), as described by Osada.^37,42^ TEM analysis of M_58_A_45_/pEGFP, M_29_A_29_B_9_/pEGFP, and M_62_A_52_B_32_/pEGFP in MilliQ water further demonstrated that copolymers with longer cationic blocks tend to form rods with shorter lengths as the N/P ratio increases from 2.5 to 5, possibly depending on a different pDNA folding in the presence of an excess of polycation chains (Figure S13).

Given that our GPPs were considered for subcutaneous (s.c.) administration, their nano- to micrometric size could favor DC uptake at the injection site and ensuing transport for lymph node homing, where antigen presentation occurs, as expected for large-sized particles (500–2000 nm).^43^

### 3.3 GPP Stability

**Figure 2:**
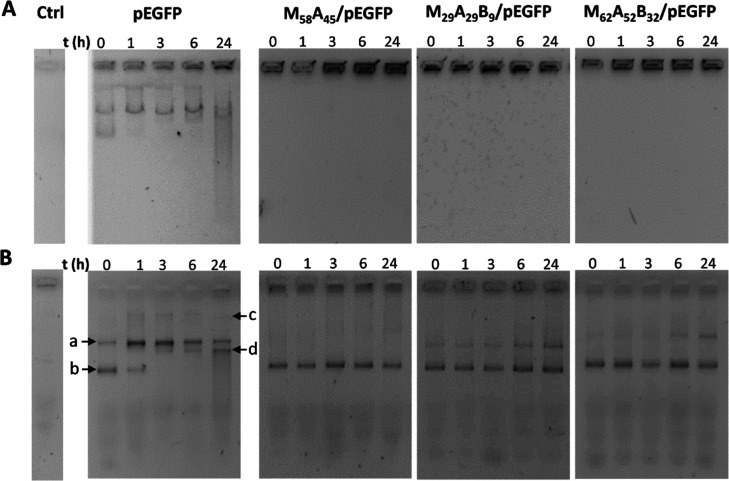
Physical (A) and chemical (B) stability of M_58_A_45_/pEGFP, M_29_A_29_B_9_/pEGFP, and M_62_A_52_B_32_/pEGFP GPPs. GPPS were incubated for 0, 1, 3, 6, and 24 h with PBS supplemented with 10% v/v FBS (vehicle) and then analyzed by agarose gel electrophoresis. Vehicle alone (Ctrl) and free pEGFP were used as controls. In the case of chemical stability analysis, prior to gel loading pEGFP, release was induced by GPP incubation for 5 min with 1.5 μL of a 10% w/v solution of SDS in water. GPPs were formulated at the N/P ratio of 2.5. Different pDNA forms are indicated by arrows. (a) Linear DNA; (b) supercoil DNA; (c) open circular; and (d) nicked.

In order to deliver an intact cargo to target cells, GPPs have to protect the loaded nucleic acids from degradation by nucleases present within the interstitial fluids.^44^ We thus tested the integrity of pDNA by gel electrophoresis after incubation of M_58_A_45_/pEGFP, M_29_A_29_B_9_/pEGFP, and M_62_A_52_B_32_/pEGFP GPPs in PBS supplemented with 10% v/v FBS at 37 °C up to 24 h. We restricted our analysis to these three GPPs as they were then used for the following *in vivo* studies. As shown in Figure 2A, all of them were stable, and no pDNA release was observed throughout the 24 h. Conversely, GPPs displayed dissimilar protection of pDNA from fragmentation. Especially, M_58_A_45_/pEGFP was the most efficient and maintained the plasmid in the supercoiled form (Figure 2B(b)) preserving pDNA from the activity of serum nucleases. M_29_A_29_B_9_/pEGFP and M_62_A_52_B_32_/pEGFP showed instead a time-dependent partial linearization (Figure 2B(a)), suggestive of a progressive plasmid attack by nucleases, which was more marked for the copolymer with the lower DP (Figure 2B). Taken together, these results indicate that M_58_A_45_/pEGFP heterocomplexes are endowed with high stability and protect pDNA from degradation.

At the same time, naked pEGFP showed expected degradation over time, with the progressive appearance of linearized (Figure 2B(a)), open circular (Figure 2B(c)), and eventually nicked DNA (Figure 2B(d)), as a result of serum nuclease activity.

### 3.4 Mannosylation Enhances pDNA Transfection in CD206-Expressing Cells

We next devised a series of experiments to test the ability of GPPs to mediate pDNA transfection in living cells using pEGFP as model cargo, monitoring EGFP fluorescence by flow cytometry and confocal microscopy. In addition, to examine the contribution of the mannosylated block as a targeting agent, we used CHO cells, that do not constitutively express the mannose receptor, and CHO-CD206^+^, stably expressing the mannose receptor, that together represent a very convenient system to discern the receptor-mediated versus the unspecific internalization of our mannosylated GPPs. Initial experiments were performed using pEGFP as model cargo and monitoring EGFP fluorescence by flow cytometry and confocal microscopy to assess cell transfection.

**Figure 3:**
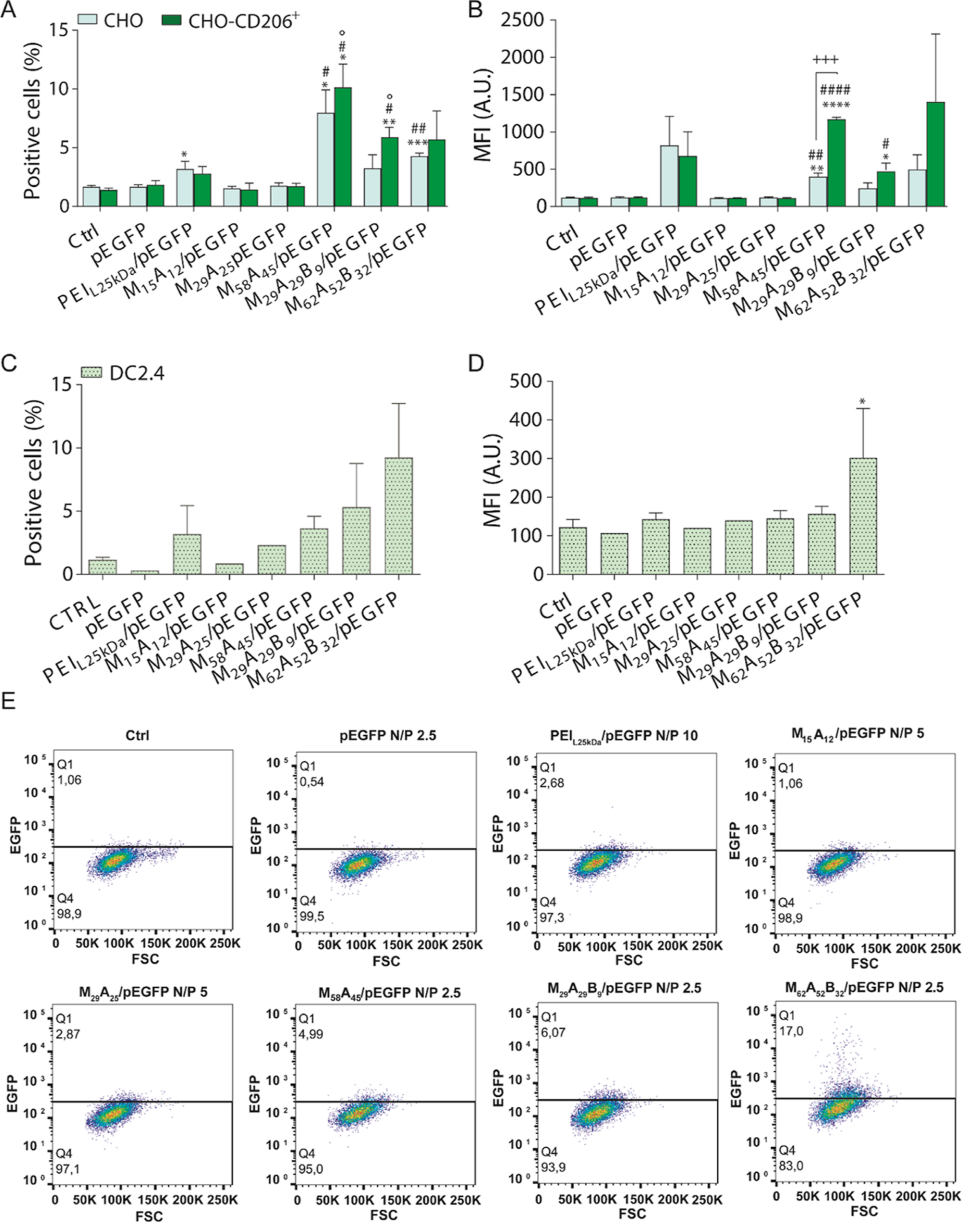
Evaluation of pEGFP TE via flow cytometry (A–E). EGFP positive cells percentage (A,C) and mean fluorescence intensity (B,D) of CHO and CHO-CD206^+^ (A,B) and DC2.4 (C,D) cells after 6 h incubation with pEGFP, PEI_L25kDa_/pEGFP at 10 N/P ratio, M_15_A_12_/pEGFP and M_29_A_25_/pEGFP GPPs formulated at the N/P ratio of 5, and M_58_A_45_/pEGFP, M_29_A_29_B_9_/pEGFP, and M_62_A_52_B_32_/pEGFP GPPs formulated at the N/P ratio of 2.5, and additional 24 h post-transfection incubation (2.5 μg mL^–1^ pEGFP concentration). Untreated (Ctrl) and pEGFP-treated cells were used as control. MFI (A.U.): mean fluorescence intensity (arbitrary unit). Data are presented as mean ± s.d. of three independent experiments performed in duplicates (except pEGFP, M_15_A_12_/pEGFP, and M_29_A_25_/pEGFP GPPs for which *N* = 1). Statistic symbols indicate *: sample vs Ctrl; ^#^: sample vs pEGFP; °: sample vs PEIL25 kDa/pEGFP. *^,#,^°*P* < 0.05; **^,##,^°°*P* < 0.01; ***^,###^*P* < 0.001; ****^,####^*P* < 0.0001.

Wild type and CHO-CD206^+^ cells were incubated for 6 h with the GPPs complexed with 2.5 μg mL^–1^ pEGFP at the N/P ratio determined before. These formulations were found to be biocompatible and did not cause cell death (Figure S5). The TE was evaluated after 24 h post-incubation with GPPs. While M_15_A_12_/pEGFP and M_29_A_25_/pEGFP GPPs mediated a negligible transfection rate comparable to that by nude pEGFP, the diblock M_58_A_45_ and the triblock GPPs produced efficient cell transfection, which resulted in comparable or even higher rate than that mediated by the standard cationic transfection agent, 25 kDa linear polyethyleneimine (PEI_L25kDa_) (Figure 3A, light green). Importantly, the percentage of fluorescent cells treated with these three GPPs was much higher in CD206^+^ cells (Figure 3A, dark green), where the rate of transfection was tangibly exceeding that of PEI_L25kDa_/pEGFP complexes formulated at the optimum N/P ratio of 10, as determined by EMSA and DLS analysis (Figure S14). Comparable results were obtained using DC2.4 (Figure 3B) and JAWSII (Figure S15) immortalized DCs, which express constitutively the CD206 receptor. Importantly, M_58_A_45_ and triblock-based GPPs also led to an increased intensity of EGFP fluorescence (mean fluorescence intensity, MFI) specifically in CHO-CD206^+^ cells (Figure 3C). This difference was particularly marked in the case of M_58_A_45_/pEGFP GPP (MFI of 401.55 ± 65.66 in CHO versus 1169.73 ± 35.68 in CHO-CD206^+^) and was clearly visible by confocal microscopy (Figure S16). On the contrary, no significant difference in EGFP MFI was observed in the two cell lines upon incubation with PEI_L25kDa_/pEGFP that lacks a CD206 targeting function. Altogether, these results indicate that the active targeting of CD206 mediated by the mannosylated outer block of our GPPs not only increased the percentage of transfected CHO-CD206^+^ cells but also enhanced their transfection yield, that is, the number of EGFP per cells. This was confirmed in DC2.4 cells where M_62_A_52_B_32_/pEGFP GPP boosted EGFP fluorescence (Figure 3D).

**Figure 4:**
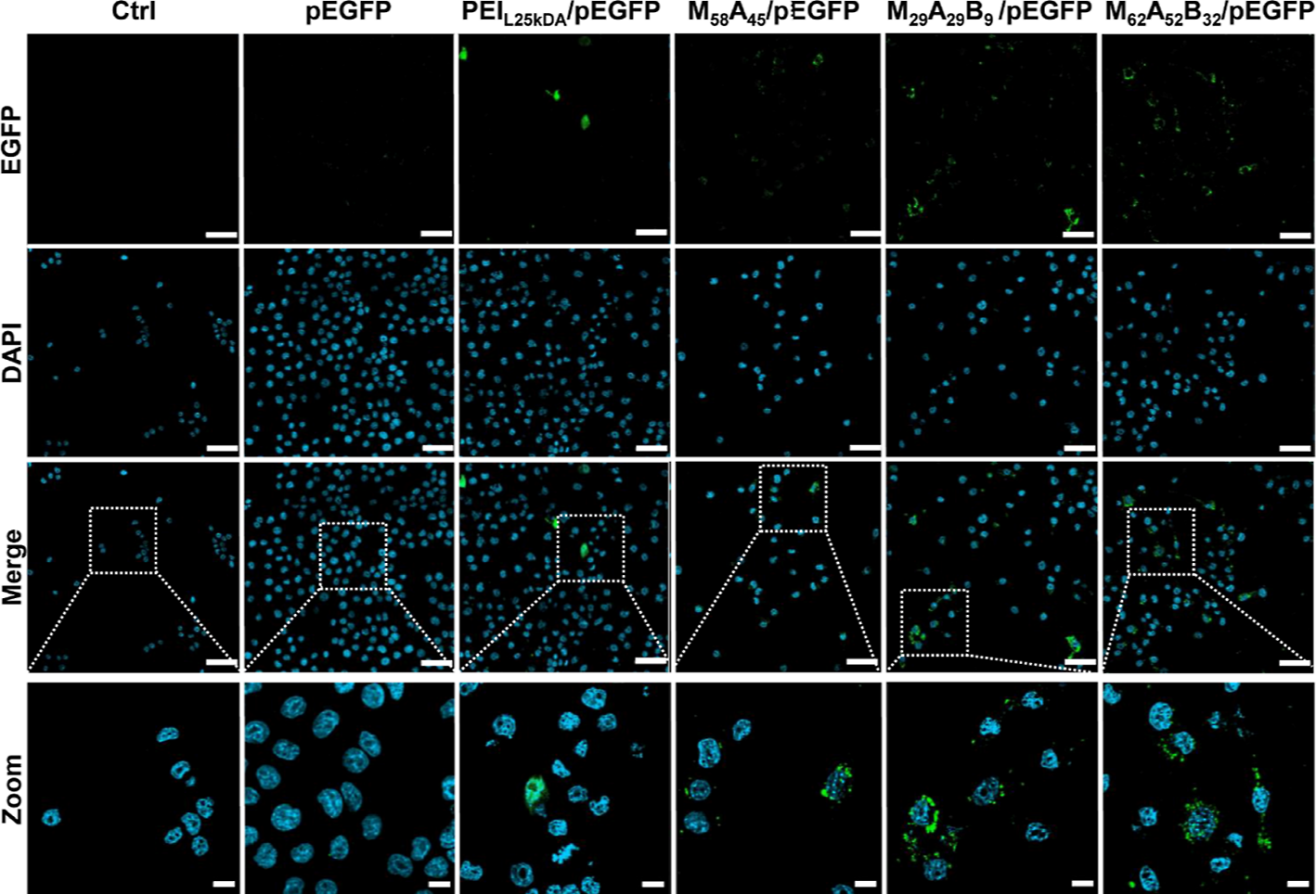
Confocal laser scanning microscopic images of DC2.4 cells transfected with pEGFP, PEI_L25kDa_/pEGFP at 10 N/P ratio, and M_58_A_45_/pEGFP, M_62_A_52_B_32_/pEGFP, and M_29_A_29_B_9_/pEGFP at the N/P ratio of 2.5. Separate channels for the zoomed images and all copolymer/pEGFP complexes are available in the Supporting Information (Figure S17). Staining: DAPI—nuclei (blue), EGFP (green). Scale bars: 50 μm (full images) and 10 μm (zoomed images).

A visual demonstration of the cell percentage and fluorescence intensity found in DC2.4 after pEGFP transfection with the different carriers is shown by the dot plots of Figure 3E, which was also assessed by confocal microscopy, confirming the cytosolic expression of EGFP (Figures 4 and S17). Considering that the cell-exogenous pDNA requires to be imported into the nucleus for its ensuing translation, it is tempting to speculate that the increased transfection rate of pEGFP complexed with our copolymer as compared to standard PEI, may be promoted, at least in part, by the (A) block containing residues of agmatine, which have been reported to favor nuclear entry.^30^

Percentage of transfection of JAWS II cells was in line with the results in CHO-CD206^+^ and DC2.4 cells (Figure S15), suggesting that transfection by diblock and triblock GPPs occurs via a mechanism of active targeting in general to CD206 expressing cells.

An important consideration emerging from these experiments is that the hydrophobic (B) block positively impacts on the TE, as indicated by the fact that M_29_A_25_ diblock does not mediate pEGFP transfection in contrast to the homologous M_29_A_29_B_9_ triblock copolymer bearing a butyl portion. This could be ascribed to the membrane disruption properties attributed to the hydrophobic block.^25^

Recently, Riley and co-workers highlighted the unsatisfactory results achieved by DNA vaccines in clinical trials mainly due to the nuclear delivery barriers^45^ and the unmet need of novel and more effective delivery platforms. In this scenario, M_58_A_45_/pEGFP, M_29_A_29_B_9_/pEGFP, and M_62_A_52_B_32_/pEGFP GPPs outperforming the PEI_L25kDa_/pEGFP complex in protein expression can be pointed as a promising platform.

### 3.5 M_58_A_45_ and M_62_A_52_B_32_ Copolymers Mediate pOVA Transfection and Antigen Presentation in Model DCs

To examine the activity of M_58_A_45_, M_29_A_29_B_9_, and M_62_A_52_B_32_ copolymers as vaccine carriers, GPPs were formulated with a plasmid encoding for chicken ovalbumin (pOVA), a model antigen-bearing immunodominant CD8^+^ (OVA_257–264_) and CD4^+^ (OVA_323–339_) epitopes allowing to characterize T-cell responses.

**Figure 5:**
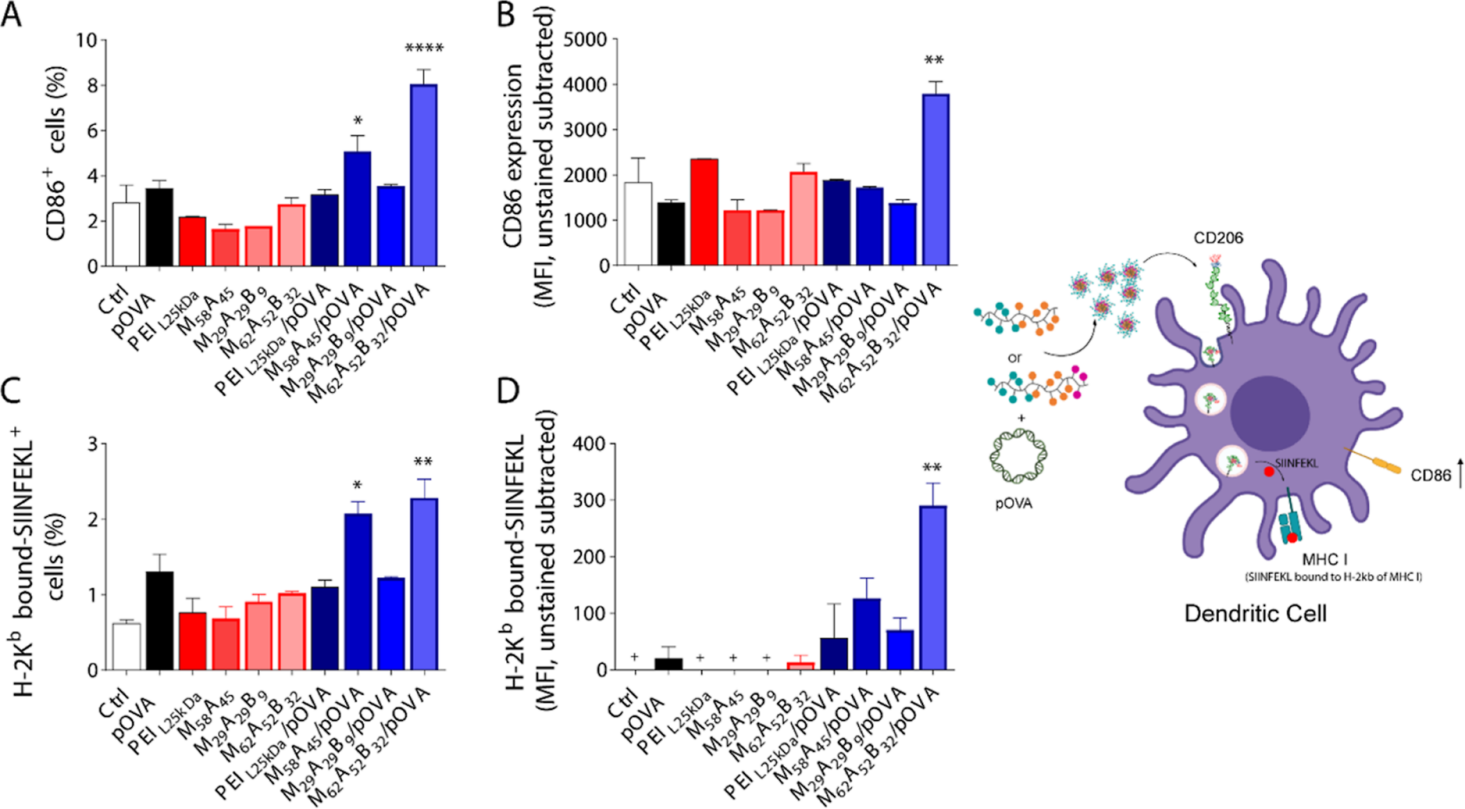
CD86 expression and H-2K^b^–SIINFEKL presentation on JAWS II mouse DCs. CD86 (A,B) or H-2K^b^–SIINFEKL (C,D) positive cells percentage (A,C) and MFI (B,D) after cell incubation with pOVA, PEI_L25kDa_, M_58_A_45_, M_29_A_29_B_9_, and M_62_A_52_B_32_ copolymers and PEI_L25kDa_/pOVA formulated at the N/P ratio of 10, and M_58_A_45_/pOVA, M_29_A_29_B_9_/pOVA, and M_62_A_52_B_32_/pOVA GPPs formulated at the N/P ratio of 2.5 (2.5 μg mL^–1^ pOVA concentration) as detected by flow cytometry. Untreated cells were used as control (Ctrl). (D) Negative values were considered as zero and labeled with a “+”. (E) CD206-mediated endocytosis of copolymer/pOVA complexes leads to increased CD86 expression and presentation of SIINFEKL antigenic peptide, derived from OVA, on MHC class I. Data are presented as mean ± s.d. (*n* = 2). Statistical significance is reported as samples vs pOVA; *p < 0.05; **p < 0.01; ****p < 0.0001.

M_58_A_45_/pOVA, M_29_A_29_B_9_/pOVA, and M_62_A_52_B_32_/pOVA GPPs were formulated at N/P ratio of 2.5, which efficiently retained pOVA, as assessed by an agarose gel retardation assay (Figure S18). Thereafter, we added the GPPs to cultured JAWS II DCs, which express high levels of the lineage DC marker CD11c (Figure S19) and assessed their activation by monitoring the membrane expression of CD86, a costimulatory molecule essential to induce T-cell stimulation. While naked pOVA did not cause any activation of JAWS cells, M_58_A_45_/pOVA and M_62_A_52_B_32_/pOVA GPPs significantly increased the percentage of CD86 expressing cells (Figure 5A). Of note, this effect was specifically due to pOVA transfection rather than to unspecific cell activation, as shown by the absence of effects using the carriers alone (no plasmid). Importantly, the PEI_L25kDa_/pOVA complex failed to induce CD86 expression in any cells (Figure 5A), emphasizing the ability of our mannosylated carriers to trigger a specific immune response compared to a carrier with broad, yet unspecific, activity. Furthermore, M_62_A_52_B_32_/pOVA also enhanced the relative amount of CD86 expression (Figure 5B), confirming previous results on superior transfection efficacy of butyl-bearing polymers.

Consistent with the higher percentage of transfected cells, M_58_A_45_/pOVA and M_62_A_52_B_32_/pOVA GPPs also displayed a higher percentage of cells presenting the ovalbumin-specific antigenic-epitope “SIINFEKL” onto MHC class I (Figure 5C). Once again, this result was accompanied by a significant increase in the amount of membrane-exposed peptides with better results for M_62_A_52_B_32_/pOVA as compared to M_58_A_45_/pOVA, while the carrier alone and PEI_L25kDa_/pOVA did not elicit any SIINFEKL expression (Figure 5D), corroborating the results obtained with CD86.

### 3.6 Nanovaccines Immuno-Mediated Tumor Growth Inhibition

Building up on our positive results of targeted transfection and successful antigen presentation in cultured DCs, we decided to test our GPPs *in vivo* on tumor-bearing mice to evaluate their potential as carriers for cancer vaccination and immunotherapy. To this aim, we took advantage of a murine tumor model based on the implant in mice of syngeneic B16 melanoma cells engineered to express chicken ovalbumin (B16-OVA). After inoculation on the mouse flank, B16-OVA grew rapidly and originated a tumor made of cells expressing OVA in their plasma membrane, thus providing a convenient model to assess the immunization efficacy of our GPPs loaded with pOVA and, at the same time, to track the tumor-specific T-cell activation and response.^46^

T-cell activation, proliferation, and differentiation, which are all needed to develop an antitumor response *in vivo*, leverage on the combined engagement of membrane T-cell receptors (TCR) and CD28 on T-cells with the MHC-peptide and CD86/CD80 couple on the membrane of APCs (Scheme 1).^47^ Our results show that M_29_A_29_B_9_ mediated potent pEGFP transfection but its performance on DC activation was modest and comparable to that of PEI_L25kDa_, suggesting that it does not represent a good candidate for *in vivo* testing. On the contrary, despite lower pEGFP transfection, M_58_A_45_ and M_62_A_52_B_32_ carriers elicited conspicuous activation of DCs by pOVA transfection, thus showing the potential to stimulate a tumor-specific T-cell response *in vivo*. Accordingly, M_58_A_45_- and M_62_A_52_B_32_-based GPPs were selected for the next *in vivo* studies.

**Figure 6:**
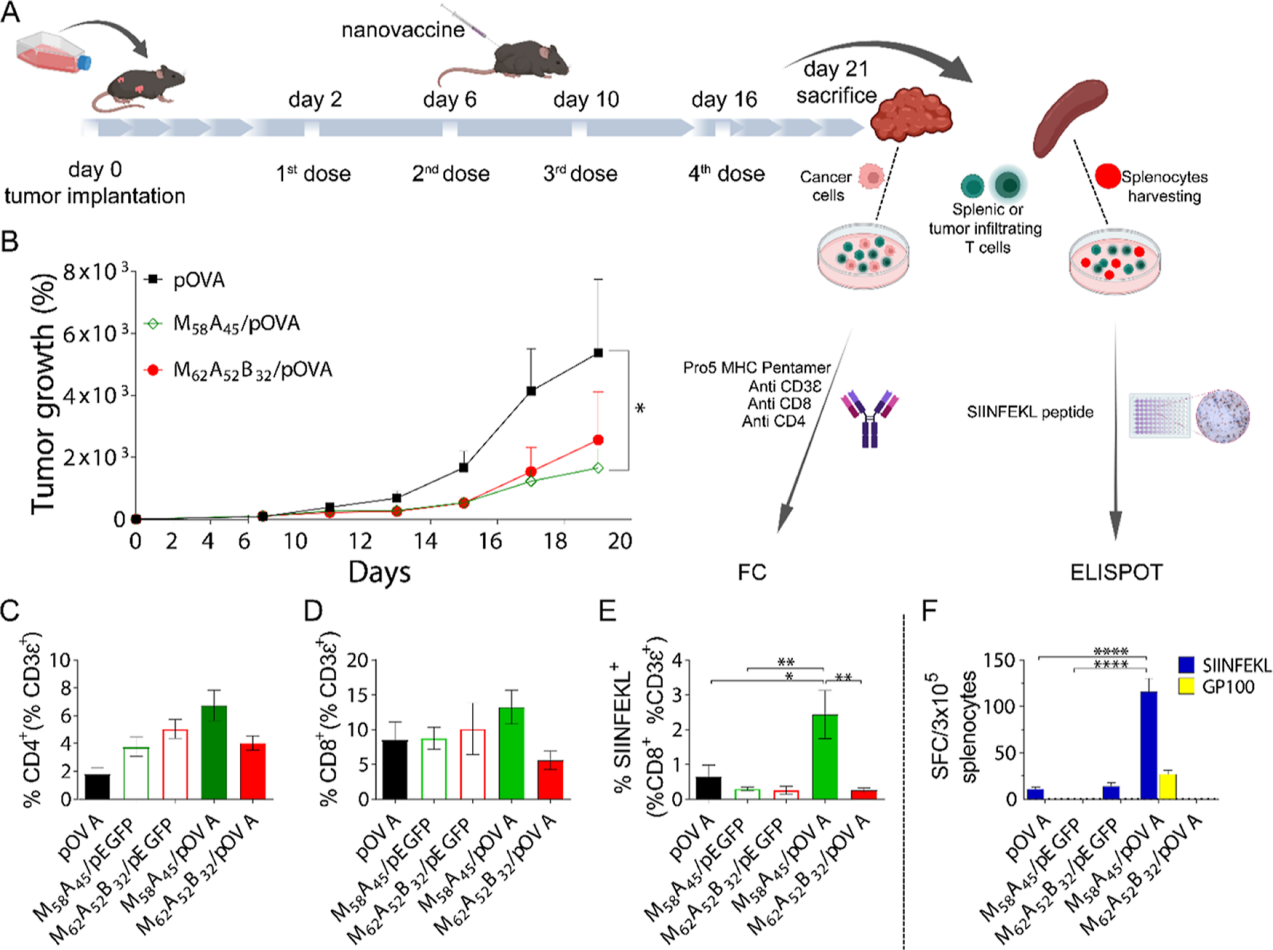
*In vivo* antitumor therapeutic efficacy. (A) Timeline for B16-OVA/B16-F1 tumor cell inoculation in C57BL/6 mice and schedule of treatment. (B) B16-OVA melanoma tumor growth after subcutaneous injection of pOVA (black), M_58_A_45_/pOVA (green), or M_62_A_52_B_32_/pOVA (red) (*N* = 4/group). Control copolymer/pEGFP polyplexes are reported in Figure S20. (C–E) Immunological analysis of T-cell population in B16-OVA tumor mass. T-cells were analyzed for their expression (reported as percentage of positive cells) of CD4^+^ (C) or CD8^+^ (D) molecules on the surface and for their ability to specifically recognize SIINFEKL bound to the MHC I molecule (E). (F) IFN-γ production by splenic T-cells isolated from B16-OVA tumor-bearing mice after their stimulation with SIINFEKL (blue bars) or gp100 (yellow bars) peptides, as revealed by ELISPOT assay. Results are reported as mean ± SEM (*n* = 4, *p < 0.05, **p < 0.01, ****p < 0.0001). FC: flow cytometry.

M_58_A_45_/pOVA and M_62_A_52_B_32_/pOVA GPPs (N/P ratio of 2.5) were subcutaneously injected into B16-OVA tumor-bearing mice at day 2, 6, 10, and 16 after tumor implantation (Figure 6A). As a control, tumor-bearing mice were also treated with either naked pOVA or M_58_A_45_/pEGFP or M_62_A_52_B_32_/pEGFP GPPs, while the same treatments were additionally applied to B16-F1 tumor-bearing mice that did not express the OVA antigen.

Tumors were monitored for 21 days and eventually harvested, dissociated, and analyzed for T-cell infiltration.

Figure 6B indicates that the M_58_A_45_/pOVA combination slowed down tumor growth, showing a 3.2-fold reduction in tumor growth as compared to that in animals treated with naked pOVA. Crucially, this effect is attributable to the efficient delivery of the pDNA encoding ovalbumin, as demonstrated either by the non-statistically significant effect of pEGFP-loaded polyplexes (negative control, Figure S20) or by the failure of M_58_A_45_/pOVA in controlling the growth of tumors induced by the inoculation of control B16-F1 melanoma cells not expressing OVA in their plasma membrane (Figure S21A).

Importantly, notwithstanding the several administrations of our complexes, treated animals did not lose weight throughout the time of treatment (Figure S22), supporting the biocompatibility and safety of our copolymers under tested conditions.

At the end of the experiment, B16-OVA tumors and spleens were collected to perform immunological analysis on the specific activation of T-cells in response to the different treatments. In agreement with the reduced tumor volume, we found a higher infiltration of CD4^+^ (Figure 6C) and CD8^+^ T-cells (Figure 6D) upon immunization with M_58_A_45_/pOVA as compared to other treatments. Importantly, CD8^+^ cells collected from M_58_A_45_/pOVA-treated mice were able to recognize the SIINFEKL-MHC I complex (Figure 6E), while this was not observed for the other treatments (2.43% of SIINFEKL-MHC I positive T-cells for M_58_A_45_/pOVA as compared to 0.66 and 0.3% of SIINFEKL reactive T-cells for pOVA and M_58_A_45_/pEGFP treatment conditions, respectively). This result indicates that M_58_A_45_/pOVA was able to mediate specific T-cell activation against the OVA antigenic epitope and suggests that the reduction in tumor volume could derive from a cytotoxic immune response developed against the tumor cells expressing the model antigen OVA in their plasma membrane. Consistently, the group treated with the M_58_A_45_/pOVA GPP also showed the highest level of IFN-γ production by splenic T-cells after re-stimulation with the SIINFEKL peptide (Figure 6F). Of note, considering that IFN-γ plays a key role in the upregulation of MHC class I and expression of co-stimulator molecules (e.g., CD86, CD40, and CD80) in DCs,^48^ thereby potentiating CD8^+^ cytotoxicity against tumor cells,^49^ these results are consistent and provide a further explanation for the strong CD8^+^ response stimulated by M_58_A_45_/pOVA.

As expected, B16-F1 tumor-bearing mice displayed no specific activation of T-cells, with both low levels of tumor infiltration and specific response in splenic T-cells following re-stimulation with the SIINFEKL peptide (Figure S21B).

Altogether, our data qualify the M_58_A_45_ copolymer as a suitable candidate for the development of novel nanovaccine platforms. Remarkably, our results were obtained with the use of M_58_A_45_ polyplexes without any adjuvants, which are expected to further potentiate the immune reaction to the transfected antigens and thus escalate the antitumor activity.

According to the *in vitro* data, we were surprised by the lack of activity *in vivo* of the M_62_A_52_B_32_/pOVA GPP. This discrepancy could be in part explained by the lower ability of M_62_A_52_B_32_ to selectively target CD206-expressing cell performance compared to that of M_58_A_45_ (Figure 3A,C), which could have been insufficient to achieve the appropriate interaction with DCs after *in vivo* administration. Moreover, displacement and stability studies (Figures 1B,2B) indicated a higher stability for M_58_A_45_-based GPP compared to all triblock copolymers, including M_62_A_52_B_32_, which also resulted in superior protection of nucleic acids by favoring pDNA supercoiling. Possibly, this could have prevented pOVA degradation by nucleases *in vivo* (Figure 2B).

In addition, the diverse size and morphology displayed by the two GPPs, with M_58_A_45_/pDNA forming rod-like particles and M_62_A_52_B_32_/pDNA assembling in bunch-like shape, may have differently affected their penetration and diffusion into the tissue as well as their cell association, as already reported by several groups.^50^

## 4 Conclusions

Nanovaccines for cancer immunotherapy have been widely used with promising results in the research setting, but clinical translation still remains a challenge, which is in part due to inefficient delivery vehicles. Thus, engineering of novel nanomaterials is actually needed to move forward novel strategies and overcome the many hurdles for cancer vaccination *in vivo*.

Here, two families of novel mannosylated polycations, diblock M_*m*_A_*n*_ and triblock M_*m*_A_*n*_B_*z*_ copolymers were synthesized by RAFT polymerization. Mannose was adopted as a targeting block to improve delivery to APCs through CD206, and agmatine was used as a condensing agent for nucleic acids. In triblock copolymers, a butyl acrylate portion was added with the aim of favoring endosomal membrane disruption and boost transfection.

All copolymers stably complexed pDNA (i.e., pEGFP and pOVA) by electrostatic interactions at relatively low N/P ratios, in the 1–5 range, forming toroid-, rod-, and spherical-shaped GPPs, depending on the copolymer length and composition. Transfection studies with copolymer/pEGFP complexes on cell cultures revealed a higher transfection performance for M_58_A_45_, M_29_A_29_B_9_, and M_62_A_52_B_32_, highlighting their potential as candidates for *in vivo* testing. This suggests that for diblock copolymers, a molecular weight threshold applies to achieve transfection, while in the presence of a butyl acrylate portion, this phenomenon was not observed. Considering the results on EGFP expression, it can be postulated that the presence of the hydrophobic segment in the triblock copolymer may facilitate the translocation of the genetic material into the cytoplasm and its accumulation in the nucleus.

M_29_A_29_B_9_-based GPP was able to transfect cells, while the corresponding M_29_A_25_ diblock copolymer lacking the butyl segment did not efficiently mediate the internalization and translation of pEGFP. Similarly, the M_62_A_52_B_32_/pEGFP complex showed higher TE when compared to the butyl-free counterpart M_58_A_45_/pEGFP and also PEI_L25kDa_/pEGFP. Further studies are warranted to elucidate their specific intracellular trafficking and transfection mechanisms.

M_58_A_45_ diblock GPPs maintained higher selectivity toward CD206-expressing cells, indicating that preferential accumulation and antigen expression in CD206^+^-APCs can be achieved via mannosylation.

*In vitro* activation studies with ovalbumin-encoding plasmid on JAWS II cells indicated that M_29_A_29_B_9_ was not suitable for promoting DC activation and antigen cross-presentation, while M_58_A_45_/pOVA and M_62_A_52_B_32_/pOVA showed superior performances than PEI_L25kDa_/pOVA.

*In vivo* experiments on tumors deriving from inoculation of B16-OVA melanoma cells revealed that M_58_A_45_/pOVA polyplexes elicited a robust and specific antitumor T-cell immunity and slowed down tumor growth.

The described work outlines an easy production of a cancer nanovaccine platform with facile preparation yet efficient nucleic acid incorporation. Future studies will come to evaluate the versatility of these delivery platforms to pack pDNA or mRNA for multiple antigens within single carriers and in combination with appropriate vaccine adjuvants, thus promoting a wide spectrum of antitumor T-cell responses for improved tumor immunotherapy.^45,51^

## Supporting Information Available

Experimental methods; synthesis of CTA and monomers; GPP cell viability studies; [CTA]/[VA-044] ratios and number of VA-044 sequential additions required for each polymerization step; NMR spectra of glycopolymers; DLS and zeta potential data of free glycopolymers and pEGFP-loaded GPPs; TEM images of GPPs and agarose gel; and DLS analyses of PEI_L25kDa_/pEGFP polyplexes; flow cytometry data for pEGFP GPPs’ TE on JAWS II cells; confocal microscopy images of CHO/CHO-CD206^+^ and DC2.4 cell transfection; gel retardation assay of pOVA-GPPs; CD11c expression on JAWS II cells under various treatment conditions; B16-OVA tumor growth for mock GPPs; B16–F1 melanoma growth and INF-γ ELISPOT results; body weight of B16-OVA or B16–F1 tumor-bearing mice; and size and PDI values of glycopolymer/pEGFP complexes (PDF)

## Notes

The data that support the findings of this study are available from the corresponding author upon reasonable request.
