# Supplementary material for: Mannosylated Polycations Target CD206+ Antigen-Presenting Cells and Mediate T-Cell-Specific Activation in Cancer Vaccination
Source: Biomacromolecules. 2022 Nov 17;23(12):5148–63. doi: 10.1021/acs.biomac.2c00993 (PMC9748946; doi:10.1021/acs.biomac.2c00993)
Supplement: Supplementary file 1 — bm2c00993_si_001.pdf [file bm2c00993_si_001.pdf]

Supporting Information

Mannosylated polycations target CD206+- antigen presenting cells and mediate T-cell specific activation in cancer vaccination

*Federica Bellato[1], Sara Feola[2,3], Gloria Dalla Verde[1], Greta Bellio,[1] Marco Pirazzini,[4] Stefano Salmaso[1], Paolo Caliceti[1], Vincenzo Cerullo[2,3], Francesca Mastrotto[1]**

[1]Department of Pharmaceutical and Pharmacological Sciences, University of Padova, Via F. Marzolo 5, 35131 Padova, IT

[2]Drug Research Program ImmunoViroTherapy Lab (IVT), Faculty of Pharmacy, Helsinki University, Viikinkaari 5E, Finland

[3]iCAN Digital Precision Cancer Medicine Flagship, FI-00014 Helsinki, Finland

[4]Department of Biomedical Sciences, University of Padova, Via Ugo Bassi 58/B, 35131 Padova, Italy.

*Corresponding author e-mail address: francesca.mastrotto@unipd.it (F. Mastrotto)

Table of content

1. Synthesis of CTA and monomers 2
   1.1 Synthesis of 4-cyano-4-(ethylsulfanylthiocarbonylsulfanyl)pentanoic acid 2
   1.2 Synthesis of D-mannopyranosyloxyethyl acrylamide (M) 2
   1.3 Synthesis of Agmatine Acrylamide (A) 6
2. Glycopolyplexes cell viability studies 7
3. Additional figures and tables 8
4. References 18

1. Synthesis of CTA and monomers

1.1 *Synthesis of 4-cyano-4-(ethylsulfanylthiocarbonylsulfanyl)pentanoic acid*

The synthesis of the RAFT agent 4-cyano-4-(ethylsulfanylthiocarbonylsulfanyl)pentanoic acid was performed in a two-step reaction according to the procedure described by Truong *et al.*[1] and already reported by us (**Scheme S*1***).[2]

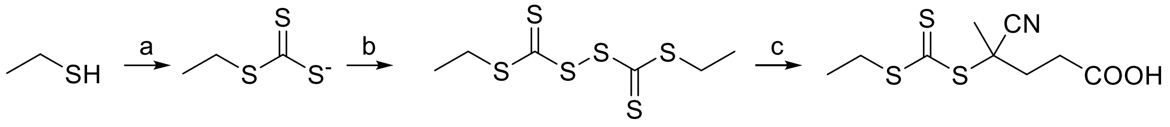

Scheme S1. Synthesis of 4-cyano-4-(ethylsulfanylthiocarbonylsulfanyl)pentanoic acid. *Reagents and conditions:* a) CS_2, basic condition, water:acetone 4:1, 0 °C; b) $K_3[Fe(CN)_6]$; c) 4,4'-azobis(cyanopentanoic acid), EtOH, 60 °C, 16 h.

Bis(ethylsulfanylthiocarbonyl)disulphide intermediate.

^{1}H NMR (400 MHz, $CDCl_3$, *δ*): 1.36 (t, 3H, C*H*$_3$, 7.5 Hz), 3.31 (q, 2H, C*H*$_2$, 7.5 Hz).

^{13}C{^{1}H}NMR (100 MHz, $CDCl_3$, *δ*): 12.5 (1C, *C*H$_3$), 32.7 (1C, *C*H$_2$), 221.4 (1C, -S*C*(S)S).

4-cyano-4-(ethylsulfanylthiocarbonylsulfanyl)pentanoic acid.

^{1}H NMR (400 MHz, $CDCl_3$, *δ*): *δ* 1.36 (t, *J* = 7.4 Hz, 3H, C*H*$_3$CH$_2$,); 1.89 (s, 3H, C*H*$_3$C), 2.34 – 2.60 (m, 2H, C*H*$_2$CH$_2$COOH), 2.65 – 2.73 (m, 2H, CH$_2$C*H*$_2$COOH), 3.33 (q, 2H, C*H*$_2$, 7.4 Hz).

^{13}C{^{1}H}NMR (100 MHz, $CDCl_3$, *δ*): δ 12.85 (1C, *C*H$_3$CH$_2$), 25.02 (1C, *C*H$_3$C), 29.48 (1C, CH$_2$*C*H$_2$COOH), 31.55 (1C, *C*H$_2$CH$_2$COOH), 33.69 (1C, CH$_3$*C*H$_2$), 46.37 (1C, CH$_3$*C*CN), 119.03 (1C, *C*N), 176.18 (1C, CH$_2$*C*OOH), 216.77 (1C, S*C*(S)S).

ESI-TOF expected m/z [M-Na]$^+$ 286.0001, found 285.9994 (100.0%).

1.2 *Synthesis of D-mannopyranosyloxyethyl acrylamide (M)*

D-mannopyranosyloxyethyl acrylamide (M) was synthesized as described by Obata *et al.*,[3] with slight modifications (**Scheme S*2***).[4]

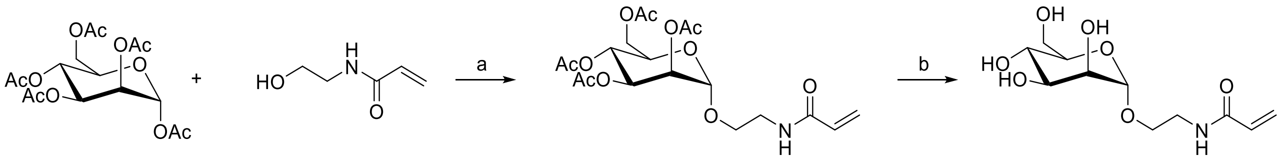

Scheme S2. Reaction scheme for the synthesis of D-mannopyranosyloxyethyl acrylamide. *Reagents and conditions:* a) BF_3Et_2O, anhydrous MeCN, 0 °C, 48 h; b) 0.02 M KOH, CH_3OH, RT, 18 h.

Synthesis of intermediate 2,3,4,6-O-tetraacetyl-D-mannopyranosyloxyethyl acrylamide. α-D-Mannose pentaacetate (10 g, 25.63 mmol) was dissolved in anhydrous acetonitrile (MeCN) (100 mL) and N-hydroxyethyl acrylamide (3.54 g, 30.76 mmol, 3.19 mL) was added under stirring. The reaction mixture was cooled down to 0 °C in an ice bath and then boron trifluoride diethyl etherate (BF_3Et_2O) (21.82 g, 153.78 mmol, 20 mL) was added dropwise over 1 h. The reaction was left under stirring at room temperature for 48 h. At scheduled times, the progress of the reaction was monitored by ^{13}C NMR analysis of samples withdrawn from the reaction solution.

At completion of the reaction, the solution was diluted with isopropanol and then the organic solvent was removed under reduced pressure. The yellowish viscous mixture was re-dissolved in dichloromethane (CH_2Cl_2) (100 mL), washed with saturated sodium bicarbonate ($NaHCO_3$) (3x100 mL) and then with deionized water (DI water) (2x100 mL). The organic layer was dried over magnesium sulfate ($MgSO_4$) and evaporated under reduced pressure. The crude product was purified by flash chromatography (Silica gel 60 Å, particle size 35-70 μm, gradient elution from 100% diethyl ether (Et_2O) to 100% ethyl acetate (EtOAc). Fractions were analyzed by thin layer chromatography (TLC) (Et_2O/EtOAc 9:1 as eluent) and the appropriate were concentrated to give the 2,3,4,6-O-tetraacetyl-D-mannopyranosyloxyethyl acrylamide (3.40 g, 7.64 mmol, 29.79% mol/mol).

^1H NMR (400 MHz, DMSO-d_6, δ): 1.93 (s, 3H, CH_3), 2.02 (s, 3H, CH_3), 2.03 (s, 3H, CH_3), 2.11 (s, 3H, CH_3), 3.37(dt, J = 7.0, 3.5 Hz, 2H, OCH_2CH_2NH), 3.52-3.60 (m, 2H, OCH_2CH_2NH), 3.97 (m, 2H, $CHCH_2COCH_3$), 4.12 (m, 1H, $CHCH_2COCH_3$), 4.89 (d, J = 1.3 Hz, 1H, C*H anomeric*), 5.10 (t, J = 9.9 Hz, 1H, C*H*), 5.15 (dd, J = 3.5, 1.6 Hz, 1H, C*H*), 5.18 (dd, J = 9.9, 3.5 Hz, 1H, C*H*), 5.60 (dd, J = 10.1, 2.2 Hz, 1H, CH=C*H*H), 6.09 (dd, J = 17.1, 2.2 Hz, 1H, CH=CH*H*), 6.25 (dd, J = 17.1, 10.1 Hz, 1H, C*H*=CH_2), 8.30 (t, J = 5.4 Hz, 1H, N*H*).

$^{13}C\{^1H\}$NMR (100 MHz, DMSO-d_6, δ): 20.37 (1C, CH_3), 20.41 (1C, CH_3), 20.42 (1C, CH_3), 20.55 (1C, CH_3), 38.84 (1C, CH_2NH), 60.22 (1C, $CHCH_2$), 62.32 (1C, CH_2CH_2NH), 65.92 (1C, *C*HOC=O), 66.74 (1C, *C*HOC=O), 68.34 (1C, *C*HOC=O), 69.17 (1C, $CHCH_2OC$=O), 97.16 (1C, *C anomeric*), 125.65 (1C, CH=CH_2), 132.05 (1C, *C*H=CH_2), 165.31 (1C, NH*C*=O), 169.95 (1C, *C*=OCH_3), 170.05 (1C, *C*=OCH_3), 170.08 (1C, *C*=OCH_3), 170.51 (1C, *C*=OCH_3).

ESI-TOF expected m/z $[M-H]^+$ 446.428, found 446.1674.

FT-IR ν = 3385, 2943, 1751, 1663, 1544, 1374, 1230, 1138, 1087, 981, 689, 601 cm^{-1}.

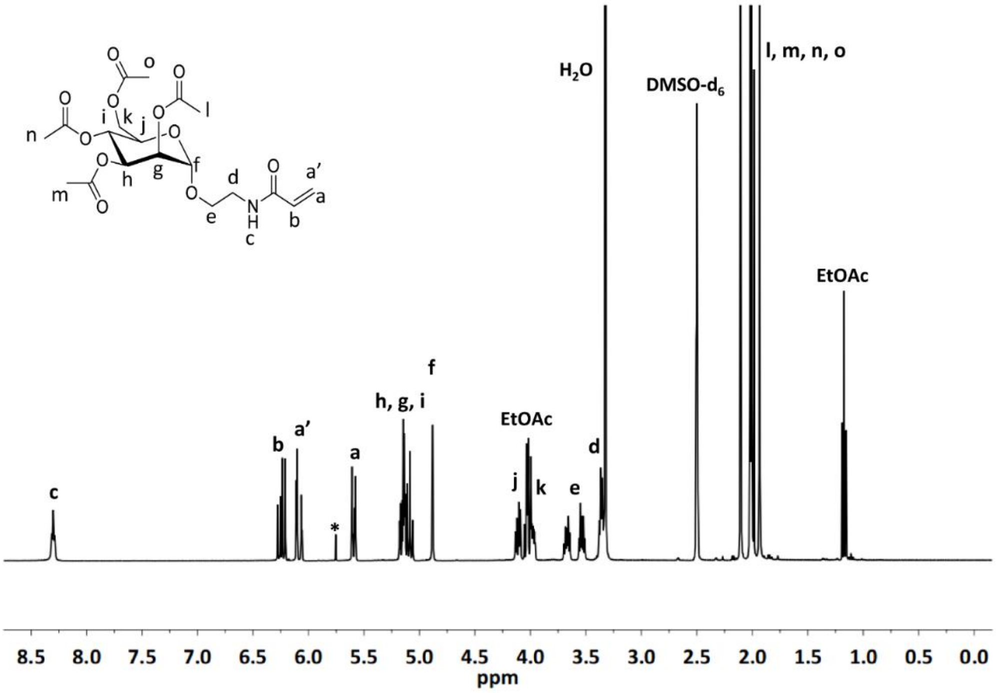

Figure S1. ^{1}H NMR spectrum in DMSO-d_6 of 2,3,4,5-O-tetraacetyl-D-mannopyranosyloxyethyl acrylamide. *: residual solvent signal - CH_2Cl_2.

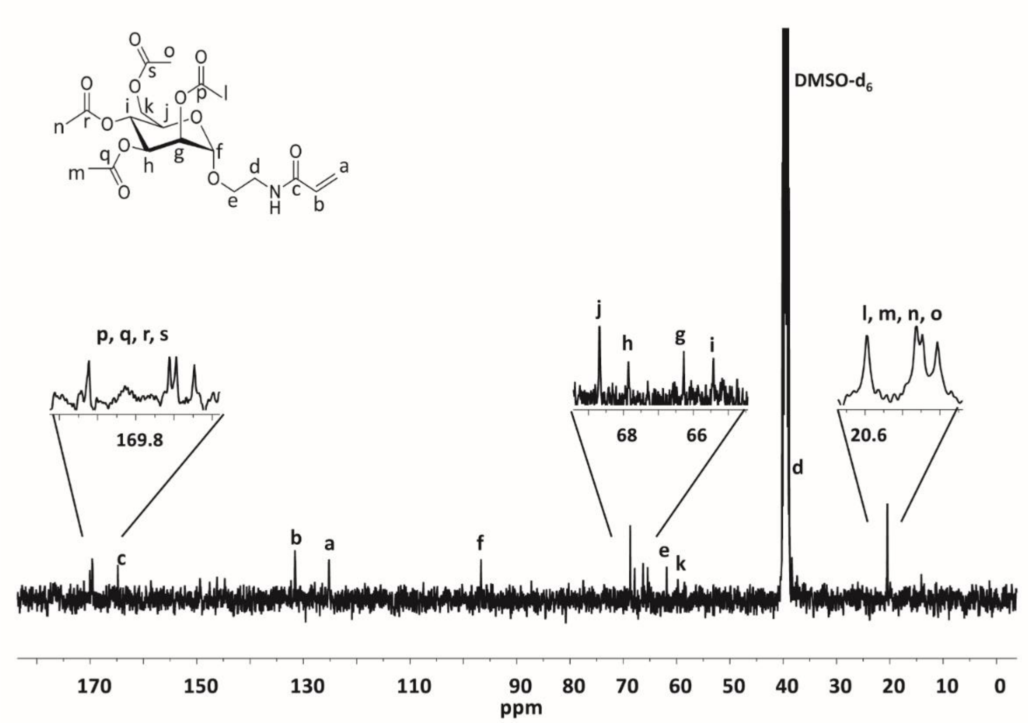

Figure S2. ^{13}C NMR spectrum in DMSO-d_6 of 2,3,4,5-O-tetraacetyl-D-mannopyranosyloxyethyl acrylamide.

Deprotection of 2,3,4,6-O-D-mannopyranosyloxyethyl acrylamide to yield D-mannopyranosyloxyethyl acrylamide. 2,3,4,6-O-tetraacetyl-D-mannopyranosyloxyethyl acrylamide (3.40 g, 7.64 mmol) was dissolved in 38 mL of a potassium hydroxide (KOH) (42.87 g, 0.764 mmol) methanolic solution. The mixture was left under stirring at room temperature for 48 h and then passed through a short pad of silica (Silica gel 60 Å, particle size 35-70 µm, eluent 100% CH_3OH). The organic solvent was removed under reduced pressure to yield D-mannopyranosyloxyethyl acrylamide as a white powder (2.055 g, 7.41 mmol, 97.11% mol/mol).

^{1}H NMR (400 MHz, DMSO-d_6, δ): 3.30 (m, 2H, CH_2NH), 3.37 (m, 2H, CH_2OH), 3.42 (m, 1H, C*H*OH), 3.45 (m, 2H, C$H_2$$CH_2$NH), 3.59 (m, 1H, C*H*OH), 3.62 (m, 1H, C*H*OH), 3.64 (m, 1H, C*H*OH), 4.47 (t, J = 5.7 Hz, 1H, O*H*), 4.58 (bs, 1H, O*H*), 4.62 (d, J = 1.4 Hz, 1H, C*H anomeric*), 4.69 (d, J = 3.9 Hz, 1H, O*H*), 4.73 (d, J = 4.1 Hz, 1H, O*H*), 5.58 (dd, J = 10.1, 2.3 Hz, 1H, CH=C*H*H), 6.08 (dd, J = 17.1, 2.3 Hz, 1H, CH=CH*H*), 6.25 (dd, J = 17.1, 10.1 Hz, 1H, C*H*=CH_2), 8.14 (t, J = 5.4 Hz, 1H, N*H*).

^{13}C{^{1}H}NMR (100 MHz, DMSO-d_6, δ): 39.58 (1C, *C*H_2NH), 61.71 (1C, *C*H_2OH), 65.83 (1C, *C*$H_2$$CH_2$NH), 67.45 (1C, *C*HOH), 70.71 (1C, *C*HOH), 71.37(1C, *C*HOH), 74.49 (1C, *C*HCH_2OH), 100.46 (1C, *C anomeric*), 125.56 (1C, CH=*C*H_2), 132.17 (1C, *C*H=CH_2), 165.17 (1C, NH*C*=O).

ESI-TOF expected m/z $[M\text{-}Na]^+$ 300.2597, found 300.1083.

FT-IR ν = 3302, 2925, 2854, 2364, 1638, 1420, 1542, 1364, 1247, 1053, 877, 654, 612 cm^{-1}.

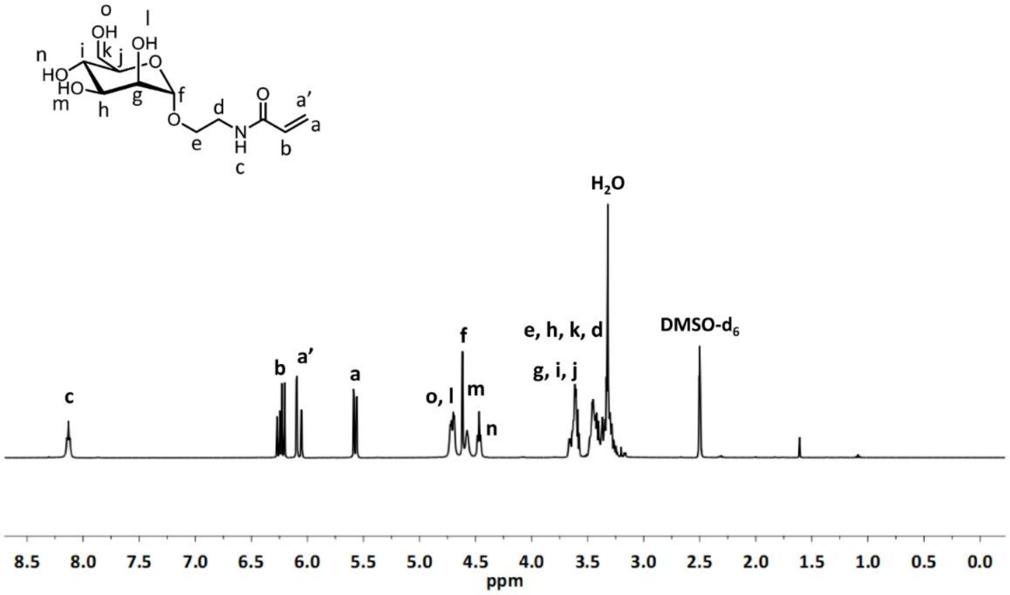

Figure S3. ^{1}H NMR spectrum in DMSO-d_6 of pure D-mannopyranosyloxyethyl acrylamide after deprotection.

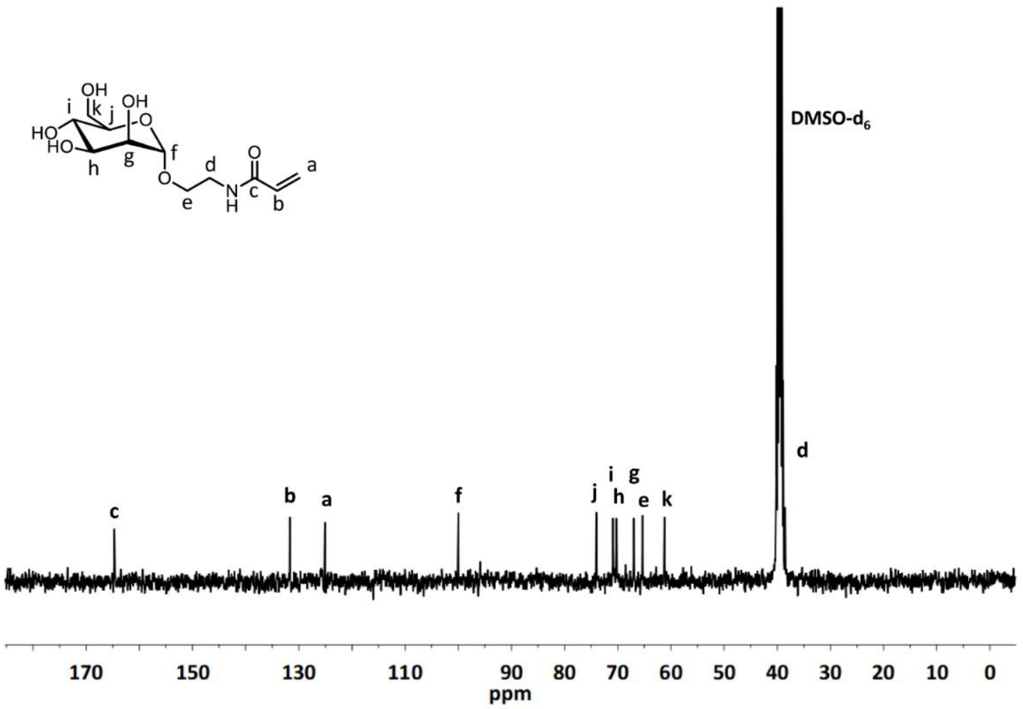

Figure S4. ^{13}C NMR spectrum in DMSO-d_6 of pure D-mannopyranosyloxyethyl acrylamide after deprotection.

1.3 Synthesis of Agmatine Acrylamide (A)

Agmatine Acrylamide (A) was synthesized according to the procedure described by Algotsson M. *et al.*,[5] with slight modifications as already reported by us.[2]

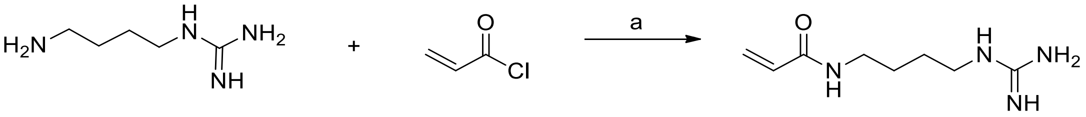

Scheme S3. Synthesis of agmatine acrylamide. *Reagents and conditions*: a) 30 mM K_2CO_3, 0 °C, 16 h.

^1H NMR (400 MHz, D_2O, δ): 1.62 (m, 4H, $NHCH_2CH_2CH_2CH_2$), 3.22-3.31 (m, 4H, $NHCH_2CH_2CH_2CH_2NH$), δ 5.77 ppm (dd, J=9.8, 1.8 Hz, 1H CH*H*=CH), δ 6.18(dd, J=17.1, 1.8 Hz, 1H, C*H*H=CH), 6.29 (dd, J=17.1, 9.8 Hz, 1H, CH_2=C*H*).

$^{13}C\{^1H\}$NMR (100 MHz, D_2O, δ): 25.41 (1C, $NHCH_2CH_2CH_2CH_2NH$), 25.77 p(1C, $NHCH_2CH_2CH_2CH_2NH$), 39.07 ppm (1C, NH*C*H_2), 40.78 ppm (1C, *C*H_2NH), 127.39 (1C, *C*H_2=CH), 130.25 (1C, CH_2=*C*H); 156.83 (1C, NH_2*C*NH); 168.32 (1C, NH*C*=O).

ESI-TOF expected m/z $[M-H^+]^{1+}$ 185.13, found 185.140.

FT-IR ν = 3314, 2875, 1648, 1605, 1264, 1087, 610, 553 cm^{-1}.

2. Glycopolyplexes cell viability studies

CHO and CHO-$CD206^+$ cells were seeded in a 96-well plate ($1x10^4$ cell $well^{-1}$) in complete medium and after overnight culture the medium was replaced with 100 µL of polymer/pEGFP glycopolyplexes suspensions prepared as described in "Electrophoretic Mobility Shift Assay". pEGFP concentrations ranged from 0.25 to 5 µg/mL and N/P ratios of 5 for $M_{15}A_{12}$/pEFGP and $M_{29}A_{25}$/pEGFP, and 2.5 for $M_{58}A_{45}$/pEGFP, $M_{29}A_{29}B_{9}$/pEGFP and $M_{62}A_{52}B_{32}$/pEGFP were used. After 24 h, cells were washed with 2x with 100 µL of PBS and the cell survival was assessed through 3-(4,5-dimethylthiazol-2-yl)-2,5-diphenyl tetrazolinium bromide (MTT) assay.[6] 180 µL of serum-free medium was added to each well followed by 20 µL of MTT solution (5 mg mL^{-1} in PBS). After 3 hours of incubation at 37 °C, the medium was removed and replaced with 200 µL of dimethyl sulfoxide (DMSO). Plates were left 15 minutes under gentle shaking and then analyzed via spectrophotometric measurement at 570 nm using ELISA plate reader. The cell viability was calculated using untreated cells as positive control (100% viability).

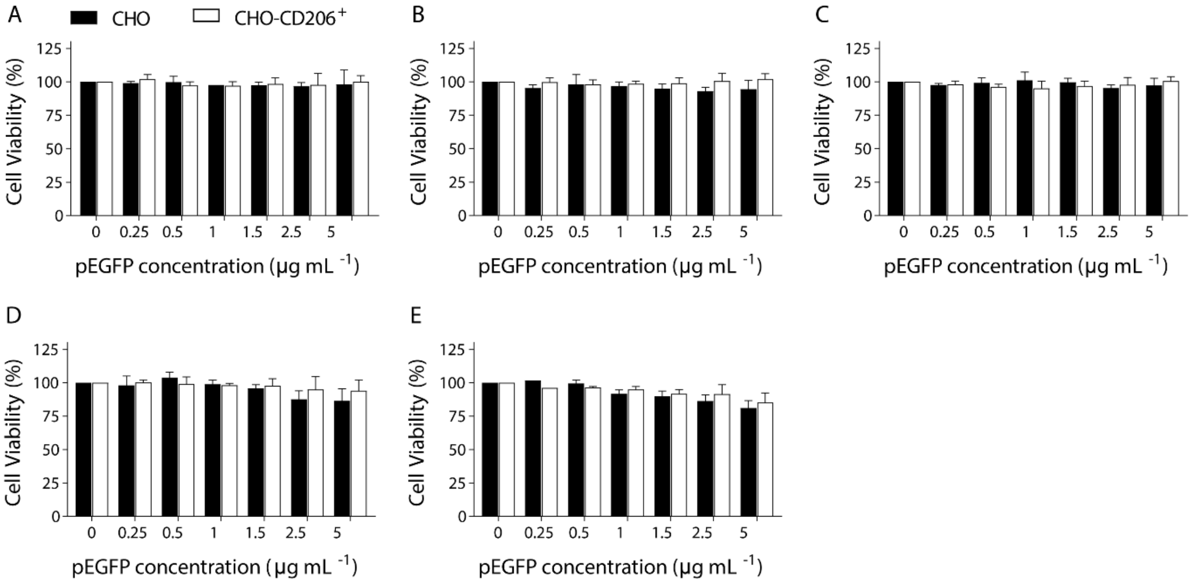

Figure S5. In vitro biocompatibility of GPPs. CHO and CHO-$CD206^+$ cells were incubated for 24 h with $M_{15}A_{12}$/pEGFP (A) and $M_{29}A_{25}$/pEGFP (B) at N/P 5, and $M_{58}A_{45}$/pEGFP (C), $M_{29}A_{29}B_{9}$/pEGFP (D) and $M_{62}A_{52}B_{32}$/pEGFP (E) at N/P 2.5 and at increasing concentration of pEGFP (0-5 µg mL^{-1}). Three independent experiments were performed in triplicate. Results are reported as mean±s.d.

3. Additional figures and table

Table S1 [CTA]:[VA-044] ratio and total µg of VA-044 used in each polymerization step of $M_{15}A_{12}$, $M_{29}A_{25}$, $M_{58}A_{45}$, $M_{29}A_{29}B_9$ and $M_{62}A_{52}B_{32}$ synthesis.

Polymer code	[CTA]:[VA-044] Molar ratio	M[a] n°; µg	A[a] n°; µg	B[a] n°; µg
$M_{15}A_{12}$	1:0.02	1; 134.9	1; 134.9	/
$M_{29}A_{25}$	1:0.02	1; 43	3; 129	/
$M_{58}A_{45}$	1:0.02	1; 29.2	2; 58.4	/
$M_{29}A_{29}B_9$	1:0.02	1; 100.5	1; 100.5	1; 100.5
$M_{62}A_{52}B_{32}$	1:0.02	1; 45	1; 45	2; 90

[a] n° indicates the number of VA-044 sequential additions required to achieve the aimed monomer conversion and the numbers are followed by the total µg of VA-044 required for the polymerization of each monomer used. M: D-mannopyranosyloxyethyl acrylamide; A: Agmatine Acrylamide B: butyl acrylate.

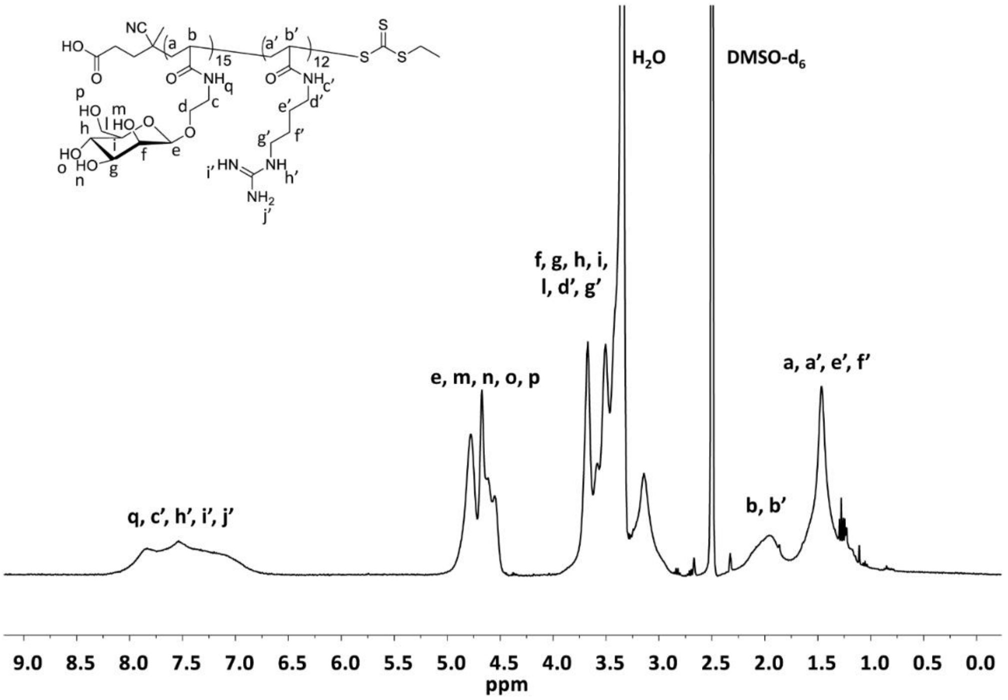

Figure S6. ^{1}H NMR spectrum in DMSO-d_6 of $M_{15}A_{12}$ after purification by dialysis and freeze-drying.

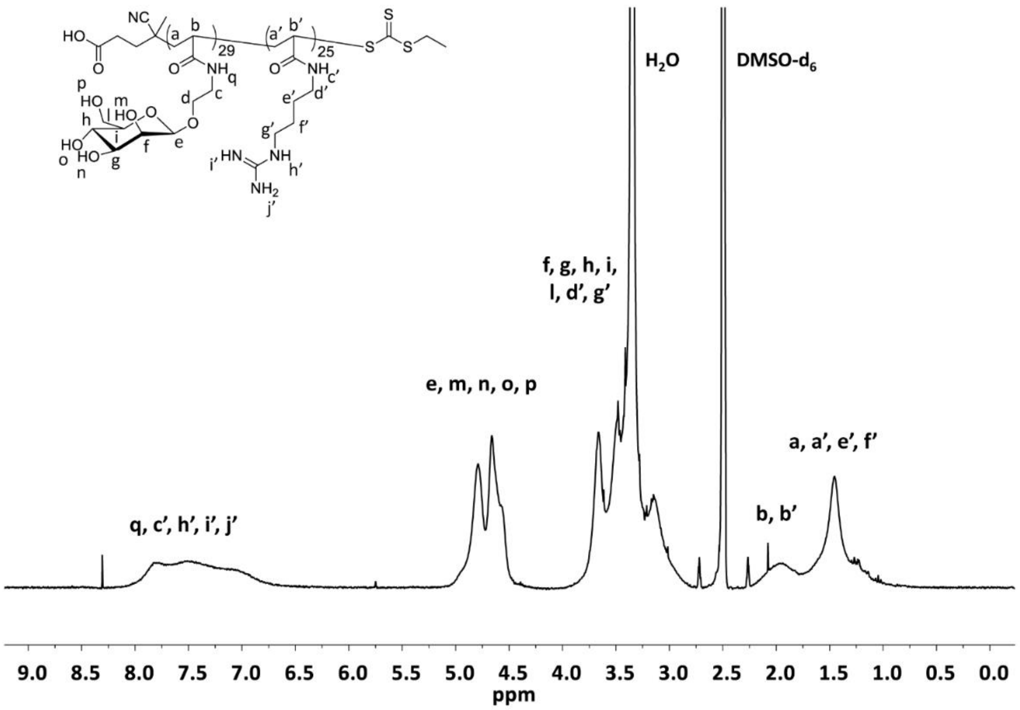

Figure S7. ^{1}H NMR spectrum in DMSO-d_6 of $M_{29}A_{25}$ after purification by dialysis and freeze-drying.

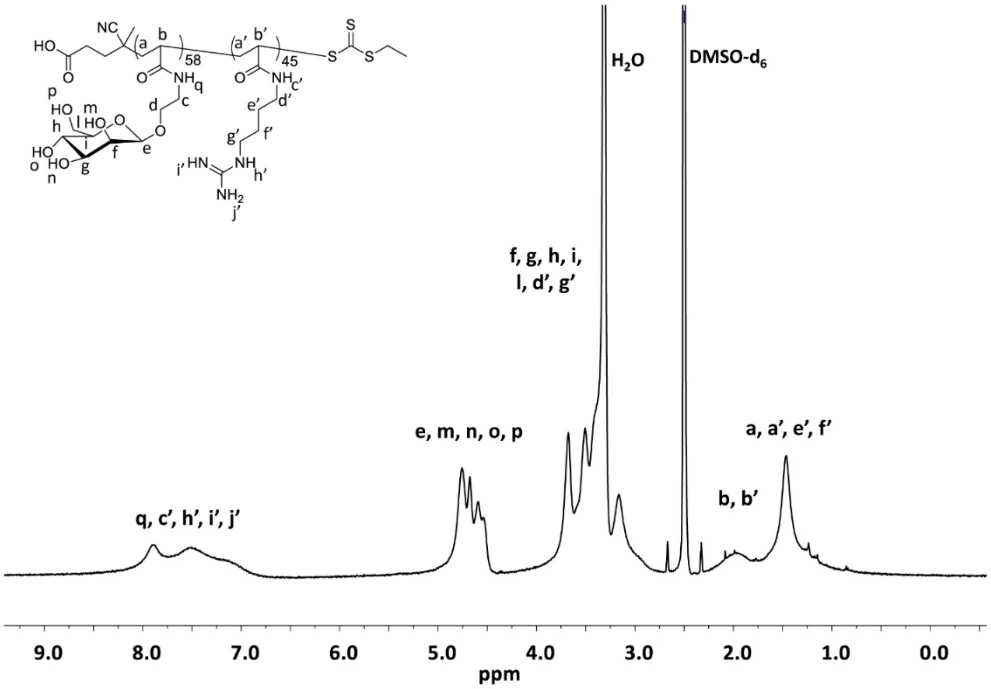

Figure S8. ^{1}H NMR spectrum in DMSO-d_6 of $M_{58}A_{45}$ after purification by dialysis and freeze-drying.

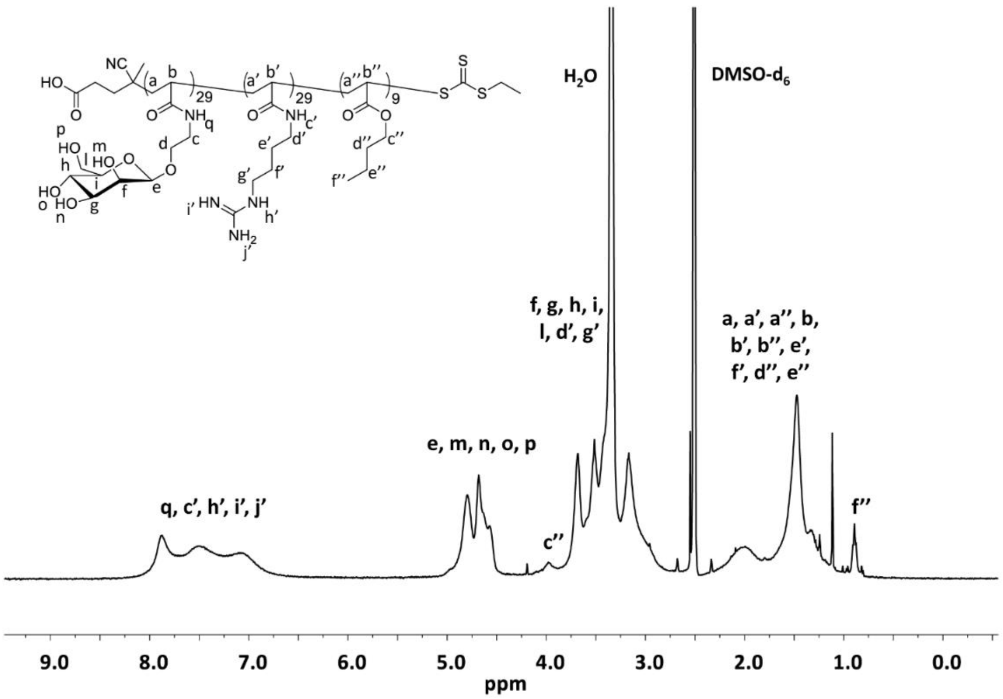

Figure S9. ^{1}H NMR spectrum in DMSO-d_6 of $M_{29}A_{29}B_9$ after purification by dialysis and freeze-drying.

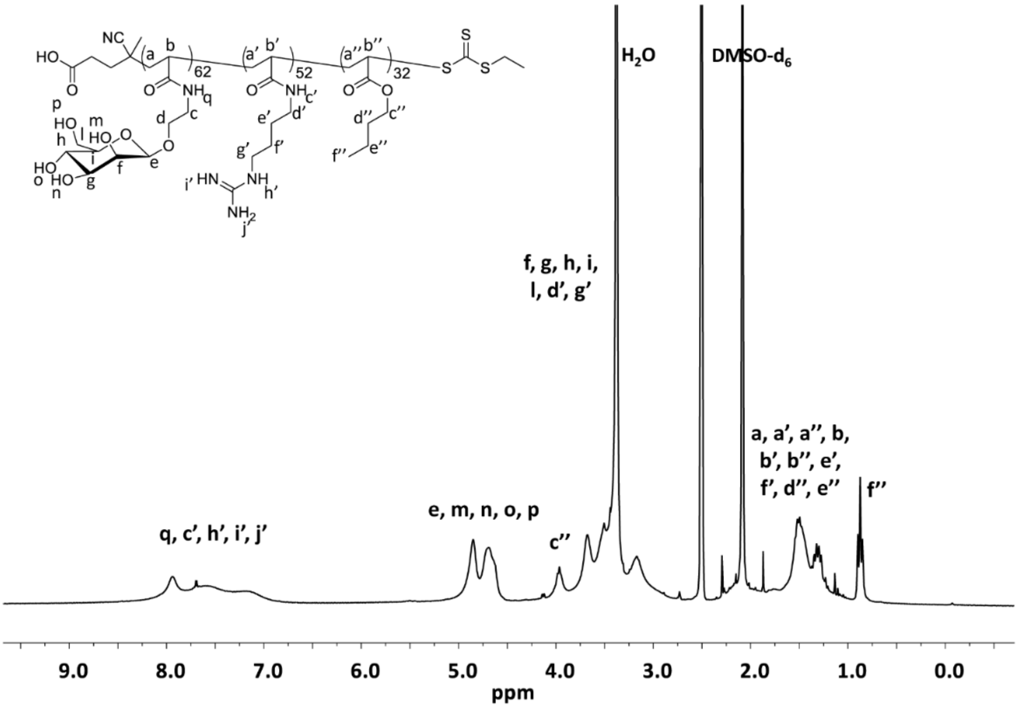

Figure S1. ^{1}H NMR spectrum in DMSO-d_6 of $M_{62}A_{52}B_{32}$ after purification by dialysis and freeze-drying.

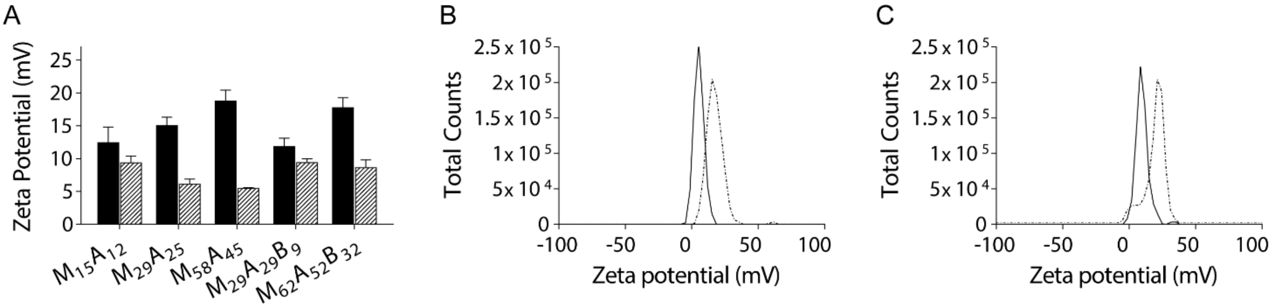

Figure S2. Histograms representation of the zeta potential values of free polymers (filled bars) and pEGFP-loaded glycopolyplexes (striped bars) in 5 mM HEPES, pH 7.4. GPPs were formulate at an N/P ratio of 5 for $M_{15}A_{12}$/pEGFP and $M_{29}A_{25}$/pEGFP, and at N/P ratio of 2.5 for $M_{58}A_{45}$/pEGFP, $M_{29}A_{29}B_{9}$/pEGFP and $M_{62}A_{52}B_{32}$/pEGFP. (A). Zeta-potential profiles of $M_{58}A_{45}$ (B) and $M_{62}A_{52}B_{32}$ (C) free polymers (dotted lines) and the corresponding pEGFP-loaded glycopolyplexes (filled lines) formulated at the N/P ratio of 2.5 in 5 mM HEPES, pH 7.4.

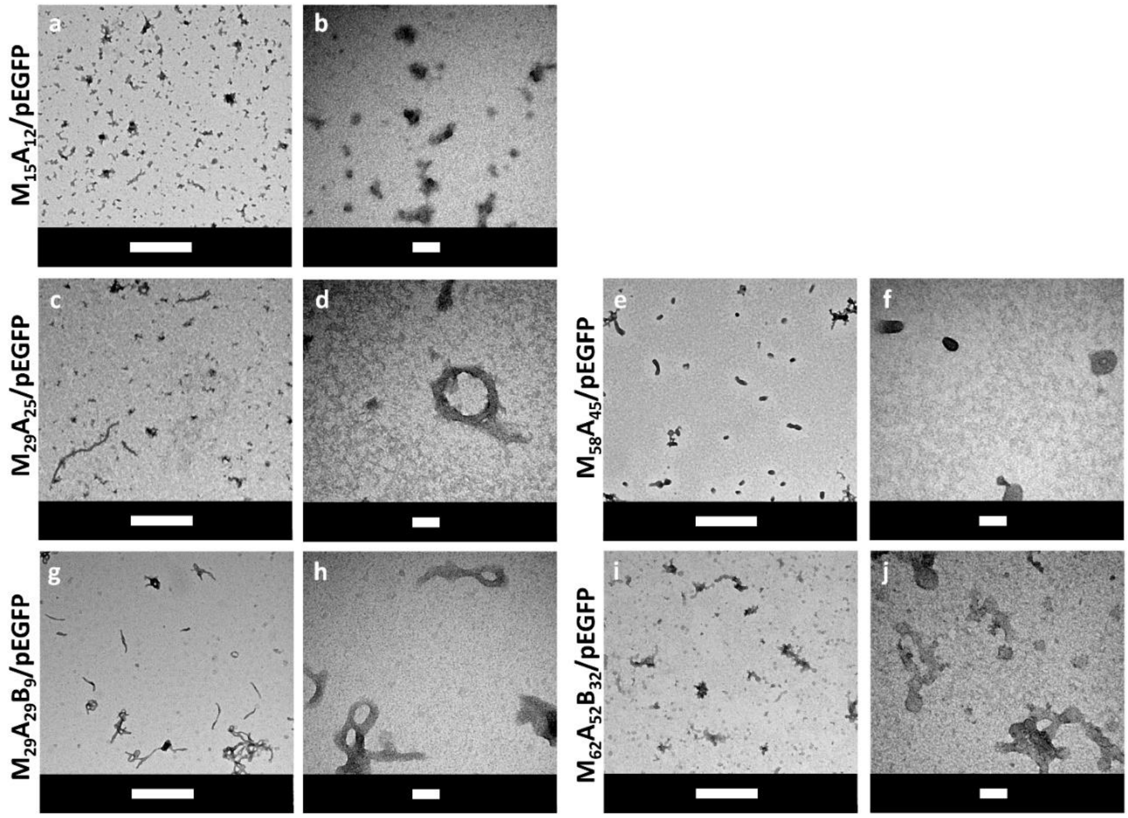

Figure S12. TEM images of $M_{15}A_{12}$/pEGFP (a,b) and $M_{29}A_{25}$/pEGFP (c,d) GPPs prepared at the N/P ratio of 5 and $M_{58}A_{45}$/pEGFP (e, f), $M_{29}A_{29}B_{9}$/pEGFP (g, h) and $M_{62}A_{52}B_{32}$/pEGFP (i, j) GPPs prepared at the N/P ratio of 2.5 in PBS. Scale bars: 500 nm (a, c, e, g, i); 50 nm (b, d, f, h, j).

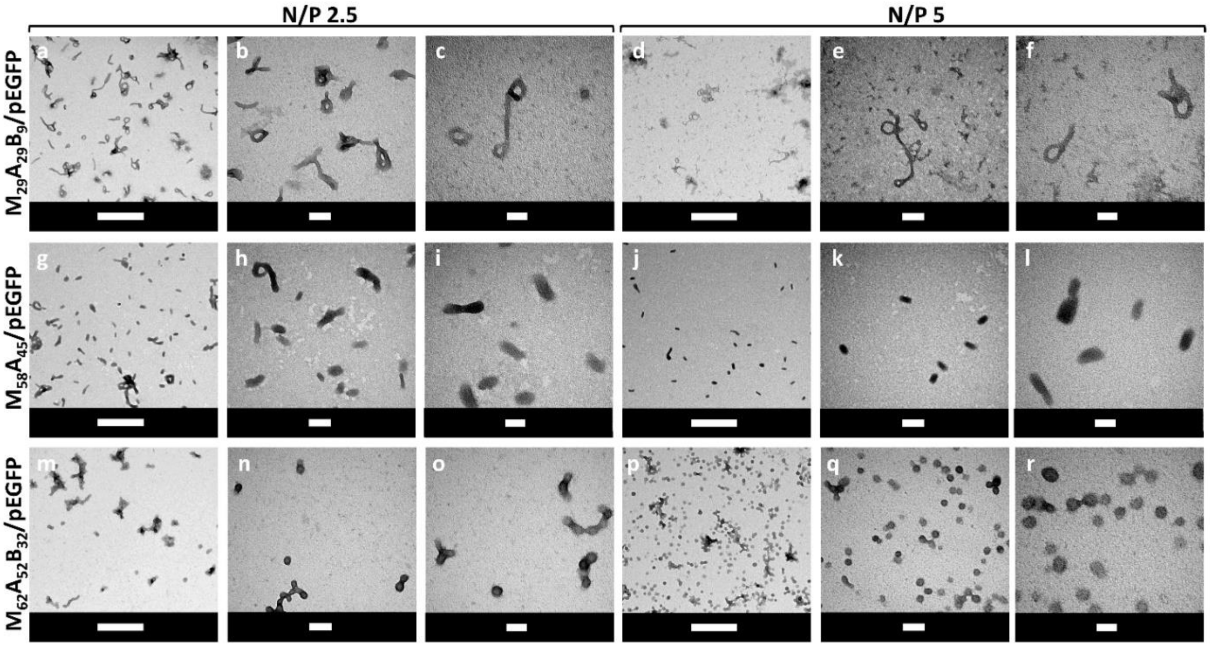

Figure S13. TEM images of $M_{29}A_{29}B_9$/pEGFP (a-f), $M_{58}A_{45}$/pEGFP (g-l), and $M_{62}A_{52}B_{32}$/pEGFP (m-r) GPPs prepared at the N/P ratio of 2.5 or 5 in milliQ water. Scale bars: 500 nm (a, d, g, j, m, p); 100 nm (b, e, h, k, n, q); 50 nm (c, f, i, l, o, r).

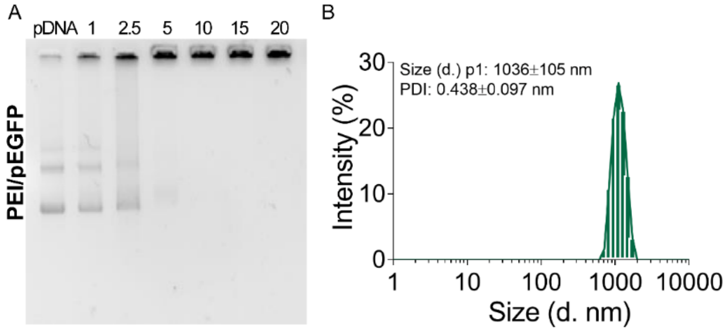

Figure S14. (A) agarose gel retardation assay of PEI_{L25kDa}/pEGFP polyplexes at various N/P ratios (1-20) and (B) hydrodynamic diameter measured by dynamic light scattering reported in intensity of PEI_{L25kDa}/pEGFP at the N/P ratio of 10.

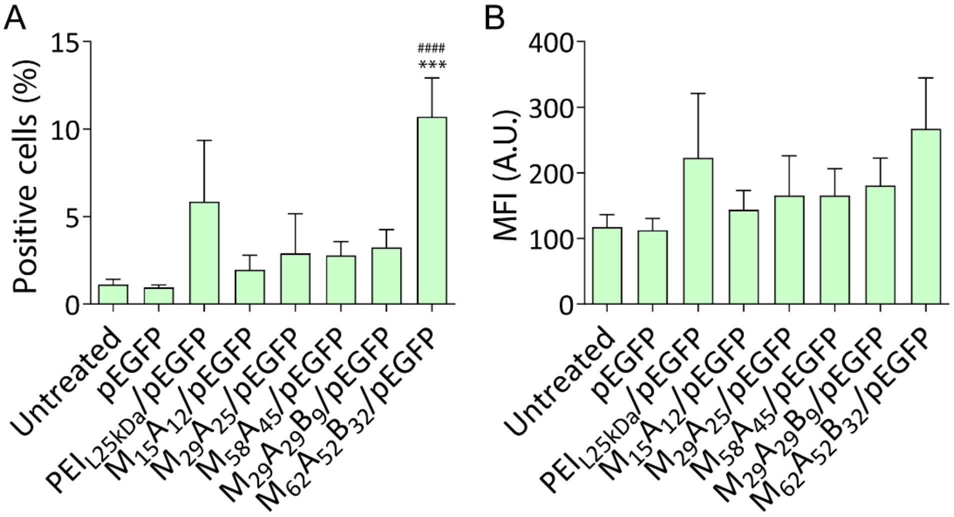

Figure S15. Evaluation of pEGFP transfection efficiency via flow cytometry (A-F) on JAWS II cells. EGFP positive cells percentage (A) and mean fluorescence intensity (B) of JAWS II cells after 6 h incubation with pEGFP, PEI_{L25kDa}/pEGFP at 10 N/P ratio $M_{15}A_{12}$/pEGFP and $M_{29}A_{25}$/pEGFP GPPs formulated at the N/P ratio of 5 and $M_{58}A_{45}$/pEGFP, $M_{29}A_{29}B_9$/pEGFP and $M_{62}A_{52}B_{32}$/pEGFP GPPs formulated at the N/P ratio of 2.5, and additional 24 h of post transfection incubation (2.5 μg mL-1 pEGFP concentration). Untreated and pEGFP-treated cells were used as control. MFI (A.U.): mean fluorescence intensity (arbitrary unit). Data are presented as mean±s.d. (n=3, N=3). *: sample vs Ctrl; #: sample vs pEGFP; ****, $^{####}$: P<0.0001.

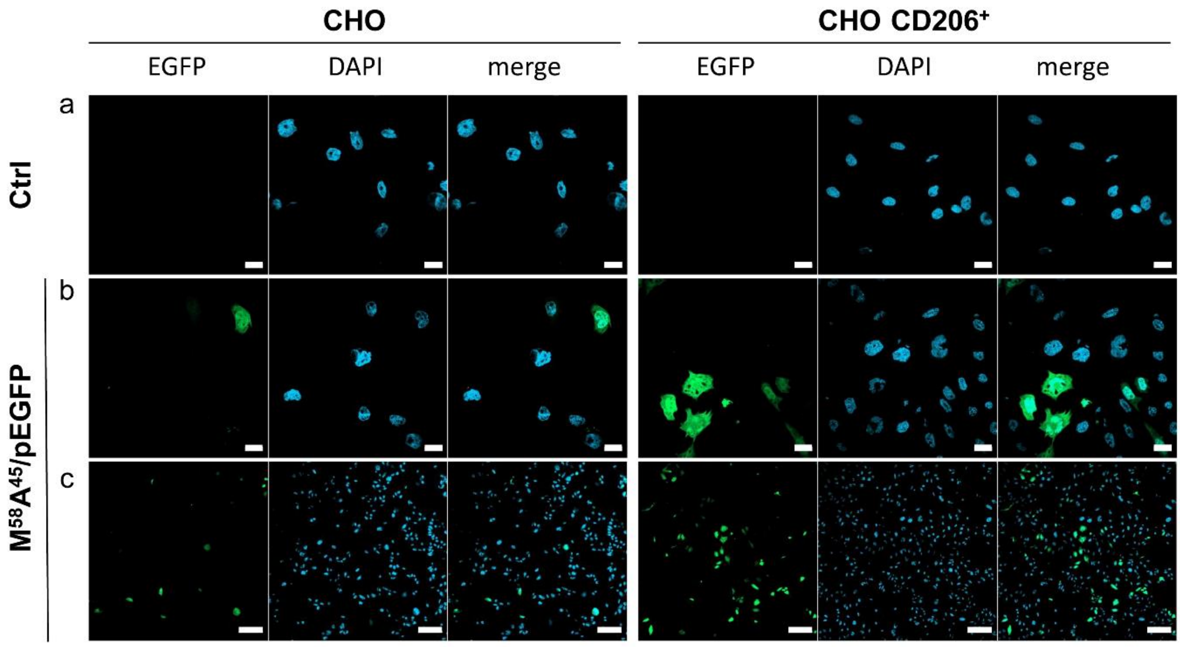

Figure S16. Confocal laser scanning microscopic images of CHO (WT) and CHO-CD206$^+$ cells after 6 h incubation with $M_{58}A_{45}$/pEGFP glycopolyplexes (2.5 µg mL^{-1} pEGFP concentration, N/P ratio 2.5) and 24 h post transfection (b, c). Untreated cells were used as control (a). Scale bars: 20 µm (a, b) and 100 µm (c).

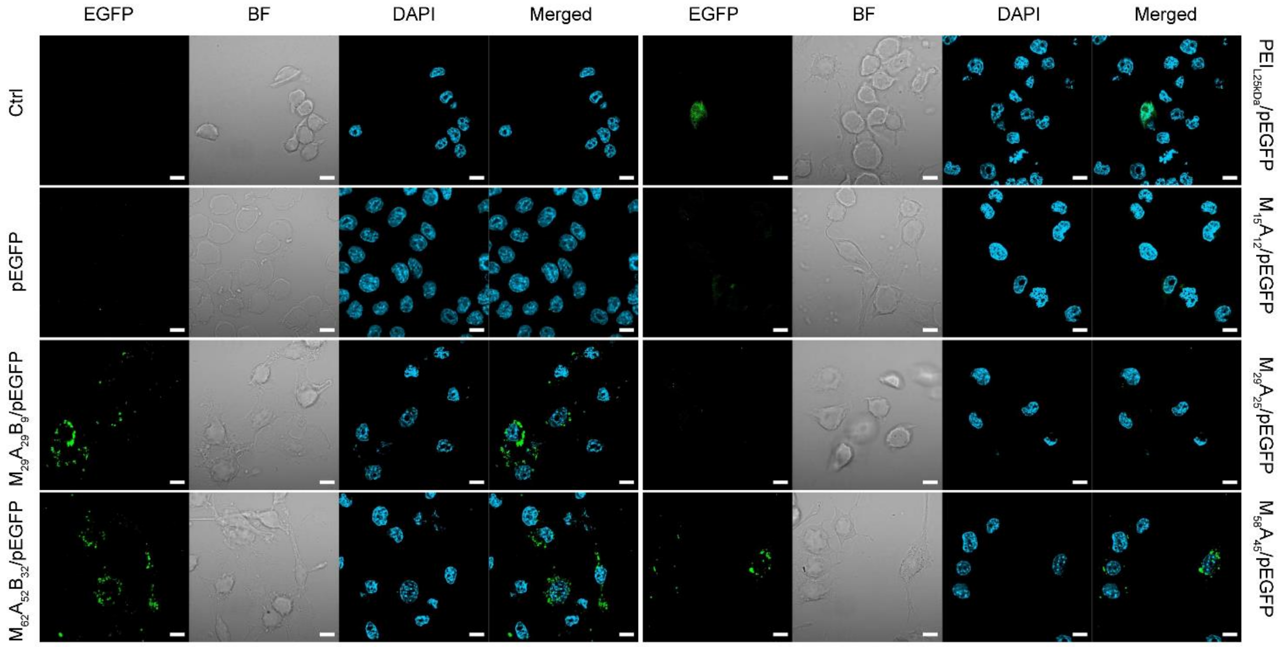

Figure S17. Confocal laser scanning microscopic images of DC2.4 cells after 6 h incubation with polymer/pEGFP glycopolyplexes (2.5 µg mL^{-1} pEGFP concentration, N/P ratio 2.5) and 24 h post transfection (b, c). Untreated cells and cells treated with pEGFP alone were used as control. Scale bar: 10 µm.

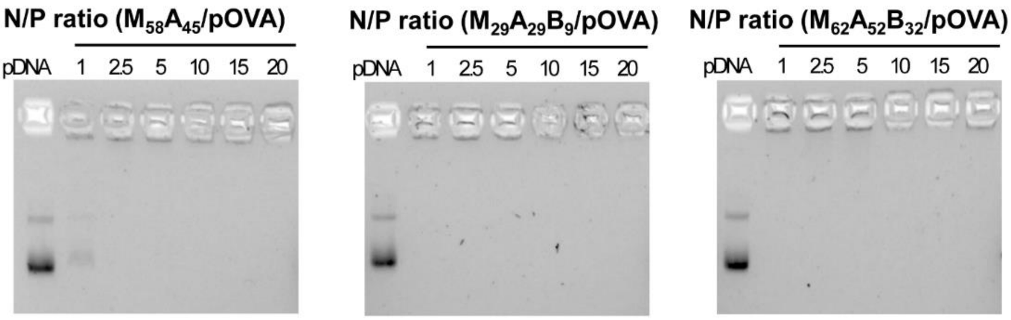

Figure S18. (A) gel retardation assay of, from left to right: $M_{58}A_{45}$/pOVA, $M_{29}A_{29}B_9$/pOVA and $M_{62}A_{52}B_{32}$/pOVA. N/P ratios in the range 0-20 were tested with gel mobility assay. Free pOVA was used as positive control.

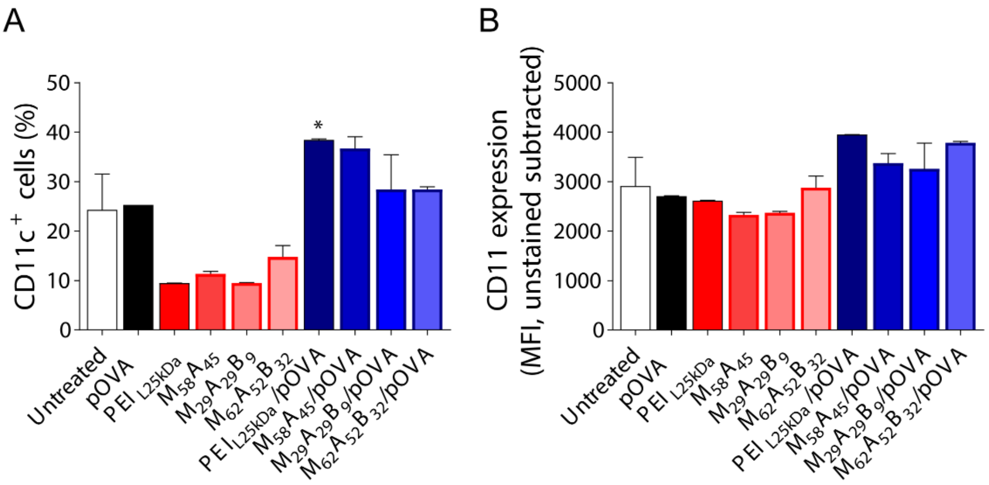

Figure S3. CD11c expression on JAWS II mouse dendritic cells. CD11c positive cells (A) and mean fluorescence intensity (MFI) (B) after cells incubation with or without pOVA, PEI_{L25kDa}, $M_{58}A_{45}$, $M_{29}A_{29}B_9$ and $M_{62}A_{52}B_{32}$ copolymers and PEI_{L25kDa}/pOVA formulated at the N/P ratio of 10, and $M_{58}A_{45}$/pOVA, $M_{29}A_{29}B_9$/pOVA and $M_{62}A_{52}B_{32}$/pOVA GPPs formulated at the N/P ratio of 2.5 (2.5 µg mL^{-1} pOVA concentration) as detected by flow cytometry. Untreated cells were used as control. Data are presented as mean±s.d. (n = 2). Symbols: *: sample vs Ctrl; *: $p < 0.05$.

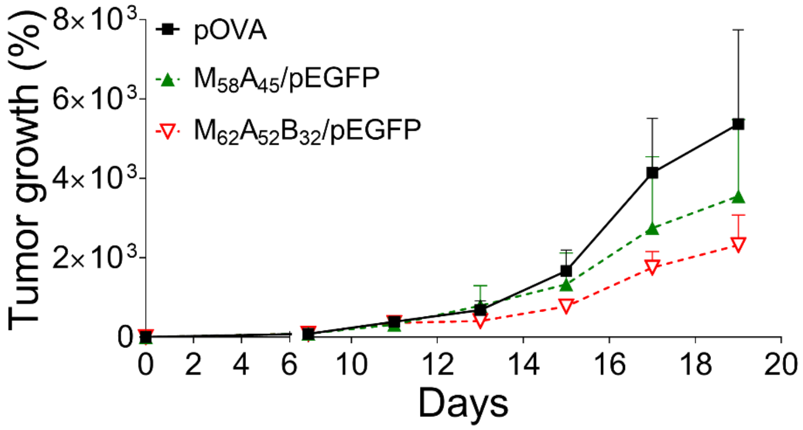

Figure S20. B16-OVA melanoma tumor growth after subcutaneous injection of pOVA (black), $M_{58}A_{45}$/pEGFP (green) or $M_{62}A_{52}B_{32}$/pEGFP (red) (N=4/group). Results are reported as mean ± SEM.

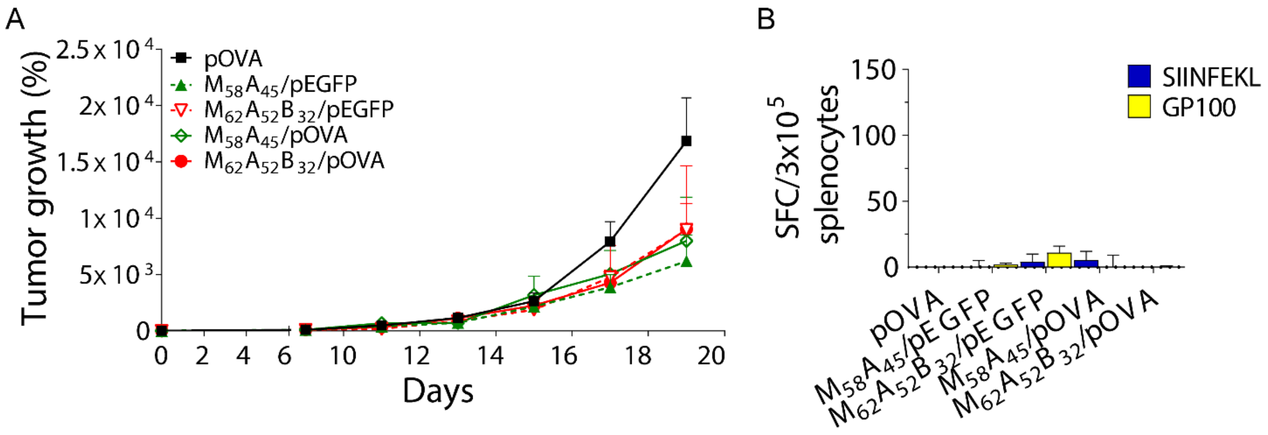

Figure S21. In vivo antitumor therapeutic efficacy of copolymer/pOVA GPPs on B16-F1 melanoma tumor model. (A) percentage of B16-F1 melanoma tumor growth after subcutaneous or peritumoral injection of pOVA, $M_{58}A_{45}$/pEGFP, $M_{62}A_{52}B_{32}$/pEGFP, $M_{58}A_{45}$/pOVA or $M_{62}A_{52}B_{32}$/pOVA. (B) IFN-γ production by splenic T-cells after restimulation with SIINFEKL (striped bars) or stimulation with gp100 (empty bars) peptides. Results are reported as mean ± SEM ($n = 4$, *$p < 0.05$, **$p < 0.01$, ***$p < 0.001$, ****$p < 0.0001$).

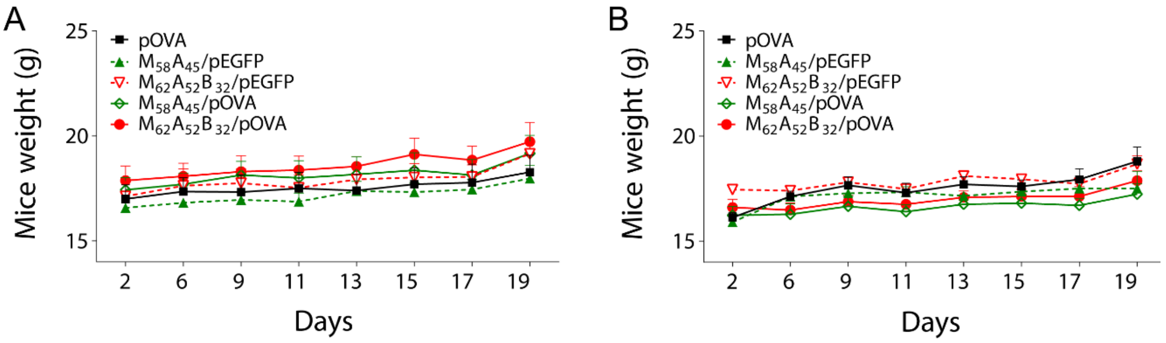

Figure S22. Animal body weight in the 17 days following treatments in B16-OVA (A) or B16-F1 (B) tumor-bearing mice.

Table S2 M_mA_n/pEGFP and $M_mA_nB_z$/pEGFP glycopolyplexes (GPPs) size reported by intensity and PDI values as detected by dynamic light scattering analysis in PBS (pDNA concentration 5 µg mL^{-1}) at the indicated N/P ratios. The theoretical number of polymer chains involved in a single molecule of pDNA condensation are also reported (copolymer/pDNA).

GPPs	N/P	Population 1 (%) (d.* nm)	Population 2 (%) (d.* nm)	PDI	Copolymer:pDNA molar ratio
$M_{15}A_{12}$/pEGFP	5	267±88 (40)	1190±445 (60)	0.550±0.03	3940:1
$M_{29}A_{25}$/pEGFP	5	262±17 (100)	/	0.363±0.053	1982:1
$M_{58}A_{45}$/pEGFP	2.5	231±14 (100)	/	0.247±0.017	525:1
$M_{29}A_{29}B_9$/pEGFP	2.5	315±36 (100)	/	0.308±0.036	815:1
$M_{62}A_{52}B_{32}$/pEGFP	2.5	54±13 (11)	316±27 (89)	0.415±0.014	455:1

*d: diameter.

4. References

1. Truong, N. P.; Dussert, M. V.; Whittaker, M. R.; Quinn, J. F.; Davis, T. P., Rapid synthesis of ultrahigh molecular weight and low polydispersity polystyrene diblock copolymers by RAFT-mediated emulsion polymerization. *Polym. Chem.* **2015,** *6* (20), 3865-3874.

2. Garofalo, M.; Bellato, F.; Magliocca, S.; Malfanti, A.; Kuryk, L.; Rinner, B.; Negro, S.; Salmaso, S.; Caliceti, P.; Mastrotto, F., Polymer Coated Oncolytic Adenovirus to Selectively Target Hepatocellular Carcinoma Cells. *Pharmaceutics* **2021,** *13* (7), 949.

3. Obata, M.; Shimizu, M.; Ohta, T.; Matsushige, A.; Iwai, K.; Hirohara, S.; Tanihara, M., Synthesis, characterization and cellular internalization of poly(2-hydroxyethyl methacrylate) bearing α-d-mannopyranose. *Polym. Chem.* **2011,** *2* (3), 651-658.

4. (a) Petch, J. E.; Gurnani, P.; Yilmaz, G.; Mastrotto, F.; Alexander, C.; Heeb, S.; Cámara, M.; Mantovani, G., Combining Inducible Lectin Expression and Magnetic Glyconanoparticles for the Selective Isolation of Bacteria from Mixed Populations. *ACS Appl. Mater. Interfaces.* **2021,** *13* (16), 19230-19243; (b) Catania, R.; Mastrotto, F.; Moore, C. J.; Bosquillon, C.; Falcone, F. H.; Huett, A.; Mantovani, G.; Stolnik, S., Study on Significance of Receptor Targeting in Killing of Intracellular Bacteria with Membrane-Impermeable Antibiotics. *Adv. Ther.* **2021,** *4* (12), 2100168.

5. Mattias Algotsson, P. B., Nicolas Thevenin Method for synthesis of acrylamide derivatives. 2007.

6. Mosmann, T., Rapid colorimetric assay for cellular growth and survival: application to proliferation and cytotoxicity assays. *J. Immunol. Methods* **1983,** *65* (1-2), 55-63.
